# Surface-modified measles vaccines encoding oligomeric, prefusion-stabilized SARS-CoV-2 spike glycoproteins boost neutralizing antibody responses to Omicron and historical variants, independent of measles seropositivity

**DOI:** 10.1128/mbio.02928-23

**Published:** 2024-01-09

**Authors:** Miguel Á. Muñoz-Alía, Rebecca A. Nace, Baskar Balakrishnan, Lianwen Zhang, Nandakumar Packiriswamy, Gagandeep Singh, Prajakta Warang, Ignacio Mena, Riya Narjari, Rianna Vandergaast, Kah-Whye Peng, Adolfo García-Sastre, Michael Schotsaert, Stephen J. Russell

**Affiliations:** 1 Department of Molecular Medicine, Mayo Clinic, Rochester, Minnesota, USA; 2 Vyriad Inc, Rochester, Minnesota, USA; 3 Department of Microbiology, Icahn School of Medicine at Mount Sinai, New York, New York, USA; 4 Global Health and Emerging Pathogens Institute, Icahn School of Medicine at Mount Sinai, New York, New York, USA; 5 Imanis Life Sciences, Rochester, Minnesota, USA; 6 Division of Infectious Diseases, Department of Medicine, Icahn School of Medicine at Mount Sinai, New York, New York, USA; 7 The Tisch Cancer Institute, Icahn School of Medicine at Mount Sinai, New York, New York, USA; 8 Department of Pathology, Molecular and Cell-Based Medicine, Icahn School of Medicine at Mount Sinai, New York, New York, USA; 9 Division of Hematology, Mayo Clinic, Rochester, Minnesota, USA; Fondazione Biotecnopolo di Siena, Siena, Italy

**Keywords:** COVID-19, prefusion spike, ferritine, nanoparticle

## Abstract

Serum titers of SARS-CoV-2-neutralizing antibodies (nAbs) correlate well with protection from symptomatic COVID-19 but decay rapidly in the months following vaccination or infection. In contrast, measles-protective nAb titers are lifelong after measles vaccination, possibly due to persistence of the live-attenuated virus in lymphoid tissues. We, therefore, sought to generate a live recombinant measles vaccine capable of driving high SARS-CoV-2 nAb responses. Since previous clinical testing of a live measles vaccine encoding a SARS-CoV-2 spike glycoprotein resulted in suboptimal anti-spike antibody titers, our new vectors were designed to encode prefusion-stabilized SARS-CoV-2 spike glycoproteins, trimerized via an inserted peptide domain, and displayed on a dodecahedral miniferritin scaffold. Additionally, to circumvent the blunting of vaccine efficacy by preformed anti-measles antibodies, we extensively modified the measles surface glycoproteins. Comprehensive *in vivo* mouse testing demonstrated the potent induction of high titer nAbs in measles-immune mice and confirmed the significant contributions to overall potency afforded by prefusion stabilization, trimerization, and miniferritin display of the SARS-CoV-2 spike glycoprotein. In animals primed and boosted with a measles virus (MeV) vaccine encoding the ancestral SARS-CoV-2 spike, high-titer nAb responses against ancestral virus strains were only weakly cross-reactive with the Omicron variant. However, in primed animals that were boosted with a MeV vaccine encoding the Omicron BA.1 spike, antibody titers to both ancestral and Omicron strains were robustly elevated, and the passive transfer of serum from these animals protected K18-ACE2 mice from infection and morbidity after exposure to BA.1 and WA1/2020 strains. Our results demonstrate that by engineering the antigen, we can develop potent measles-based vaccine candidates against SARS-CoV-2.

**IMPORTANCE:**

Although the live-attenuated measles virus (MeV) is one of the safest and most efficacious human vaccines, a measles-vectored COVID-19 vaccine candidate expressing the SARS-CoV-2 spike failed to elicit neutralizing antibody (nAb) responses in a phase-1 clinical trial, especially in measles-immune individuals. Here, we constructed a comprehensive panel of MeV-based COVID-19 vaccine candidates using a MeV with extensive modifications on the envelope glycoproteins (MeV-MR). We show that artificial trimerization of the spike is critical for the induction of nAbs and that their magnitude can be significantly augmented when the spike protein is synchronously fused to a dodecahedral scaffold. Furthermore, preexisting measles immunity did not abolish heterologous immunity elicited by our vector. Our results highlight the importance of antigen optimization in the development of spike-based COVID-19 vaccines and therapies.

## INTRODUCTION

For the second year since severe acute respiratory syndrome coronavirus (SARS-CoV-2) was first identified, coronavirus disease 19 (COVID-19) ranked as the third leading cause of death after heart disease and cancer (1). The number of lives taken globally by COVID-19 now exceeds more than 6.9 million, and the number of cases is above 771 million. The pandemic has disrupted lives across the globe and triggered the deepest recession since World War II (2). Although highly immunogenic and efficacious COVID-19 vaccines have been deployed, the continual emergence of immune-evasive variants of SARS-CoV-2 combined with the waning efficacy of SARS-CoV-2 vaccines still represents a major global health challenge (3–6).

Similar to other coronavirus infections, SARS-CoV-2 infection is mediated by homotrimeric class-I membrane-bound viral spike (S) proteins, which comprise an S1 domain containing the receptor-binding domain (RBD) that mediates attachment to the host cell, as well as an S2 domain containing the fusion peptide that initiates fusion with the host cell membrane (7, 8). Due to its critical involvement in the initiation of virus infection, the S protein is the major target of neutralizing antibodies (nAbs) and the antigen of choice in vaccine development (9). Of the currently approved or authorized SARS-CoV-2 vaccines, four employ two proline substitutions in the S2 domain to prevent its refolding. This prefusion-stabilized construct, referred to as S-2P, is the basis for the Pfizer-BioNTech and Moderna mRNA-based vaccines, the Janssen/J&J adenovirus 26 (Ad26)-based vaccine and the Novavax subunit-based vaccine, and was premised on homologous positions found in the MERS-CoV spike, resulting in higher titers of nAbs than those elicited by the wild-type spike protein (10). A fifth SARS-CoV-2 vaccine formulation, ChAdOx1-S, employs a membrane-anchored wild-type spike protein that sustains a trimeric prefusion conformation (11). Additional efforts to design spike-based vaccines also involve stabilizing the prefusion conformation of the spike ectodomain [reviewed in references (12, 13)]. One of the most promising antigens, HexaPro, which comprises six prolines, exhibits higher expression levels and resistance to heat and physical stress than S-2P (14) and is the antigen of choice in a Newcastle disease virus (NDV)-vectored COVID-19 vaccine candidate that is currently undergoing a phase II/III clinical trial (ClinicalTrials.gov: NCT05354024), as well as in other SARS-CoV-2 spike subunit vaccines (15, 16).

Most of the current first-generation vaccines rely on the early pandemic spike protein identified from the Wuhan-1 isolate. However, several mutations have accumulated in the spike protein, resulting in the emergence of variants of concern (VOCs) (17, 18). Of particular interest is the Omicron variant of SARS-CoV-2, which possesses extensive capabilities to escape from the neutralizing immunity elicited by mRNA-based vaccines (19–21) and has reignited debate over the need for booster vaccine doses or reformulated vaccines (22, 23). While a third or fourth vaccination dose restores the neutralization of Omicron and reduces COVID-19 severity in the short term, the currently used booster approach is unsustainable, warranting the development of vaccines that promote more durable immunity (19–21).

The rapid development of multiple COVID-19 vaccines has been crucial in curbing the ongoing pandemic; nevertheless, there are several limitations. mRNA vaccines are expensive and difficult to transport due to the freezing requirements. Although adenovirus vector-based vaccines have greater stability than mRNA vaccines and have no freezing requirements, the Food and Drug Administration (FDA) and the Centers for Disease Control and Prevention have restricted the use of Ad26.COV2 in the United States due to the rare yet serious adverse effect of thrombotic events with thrombocytopenia (24). A similar risk has been identified with mRNA vaccines, with reports noting instances of myocarditis, pericarditis, and thrombocytopenia (25–28). As a result, the development of other vaccine platforms and strategies that can elicit an enduring immune response with an acceptable safety profile is highly desirable.

The live-attenuated measles virus (MeV) vaccine is a highly attractive vectored vaccine since it has a proven track record of safety and efficacy in humans and is known to induce robust B- and T-cell responses that persist for an extended period of time, with a reported measles-specific antibody half-life of 3,014 years (29–31). This enduring immune protection has been attributed to the efficient replication and dissemination of MeV within lymphoid tissue, followed by the persistence of MeV RNA after the elimination of the infectious virus (32, 33). Consequently, a MeV-vectored vaccine holds the potential to elicit long-lasting immune responses against a diverse range of antigens. Indeed, the live-attenuated MeV vaccine has been engineered as a vectored vaccine against a variety of pathogens (34, 35), with a MeV-based vaccine candidate against Chikungunya demonstrating promising results in a phase II clinical trial (36). Efforts have also been made to use MeV-based vaccines for SARS-CoV-2 (37–39), including preclinical candidates based on the membrane-anchored wild-type spike protein (38) or the prefusion-stabilized spike protein (37). Another construct used a secreted form of S-2P with a self-trimerizing “foldON” domain replacing the transmembrane and cytoplasmic domains of the spike protein (39). However, the clinical development of a corresponding MeV-based SARS-CoV-2 vaccine candidate (V592) was recently discontinued due to low seroconversion rates, particularly among individuals with prior measles immunity (40, 41). It remains unclear at this time what role the design and oligomerization state of the spike may play in the magnitude and breadth of the elicited immune response.

Here, we aimed to generate a MeV-vectored vaccine that could induce high levels of SARS-CoV-2-neutralizing antibodies. To address the potential hindrance of preexisting anti-measles antibodies on vaccine efficacy, we utilized a MeV-based vaccine with an extensively modified surface (42). Next, we evaluated the immunogenicity of different MeV-vectored COVID-19 vaccine candidates expressing genetically modified SARS-CoV-2 spike ectodomain constructs. Our findings indicate that artificial trimerization of the SARS-CoV-2 spike protein is essential for eliciting a robust nAb response in MeV-susceptible receptor-transgenic, type-I interferon (IFN) receptor-deficient (IFNAR^−/−^-CD46Ge) mice. Scaffolding the trimeric SARS-CoV-2 spike protein onto the homododecameric neutrophil-activating protein (NAP) from *Helicobacter pylori* resulted in significantly higher production of nAbs than the use of the unscaffolded trimeric spike. Furthermore, a MeV/COVID-19 vaccine candidate encoding a historical Wuhan spike glycoprotein (GP) elicited robust production of nAbs against historical SARS-CoV-2 variants, but titers against the Omicron lineage were lower. An Omicron-matched MeV/COVID-19 booster increased the nAb responses against both Omicron and historical variants. Finally, our results demonstrate that serum antibodies induced in IFNAR^−/−^-CD46Ge mice by the MeV/COVID-19 vaccine candidate can provide protection against COVID-19 in K18-hACE2 mice after infection with former and current SARS-CoV-2 variants. These findings will contribute to the continued development of MeV/COVID-19 vaccine candidates and therapies.

## RESULTS

### Only the full-length SARS-CoV-2 spike elicits pseudovirus-neutralizing antibodies in IFNAR^−/−^-CD46Ge mice

**Fig 1:**
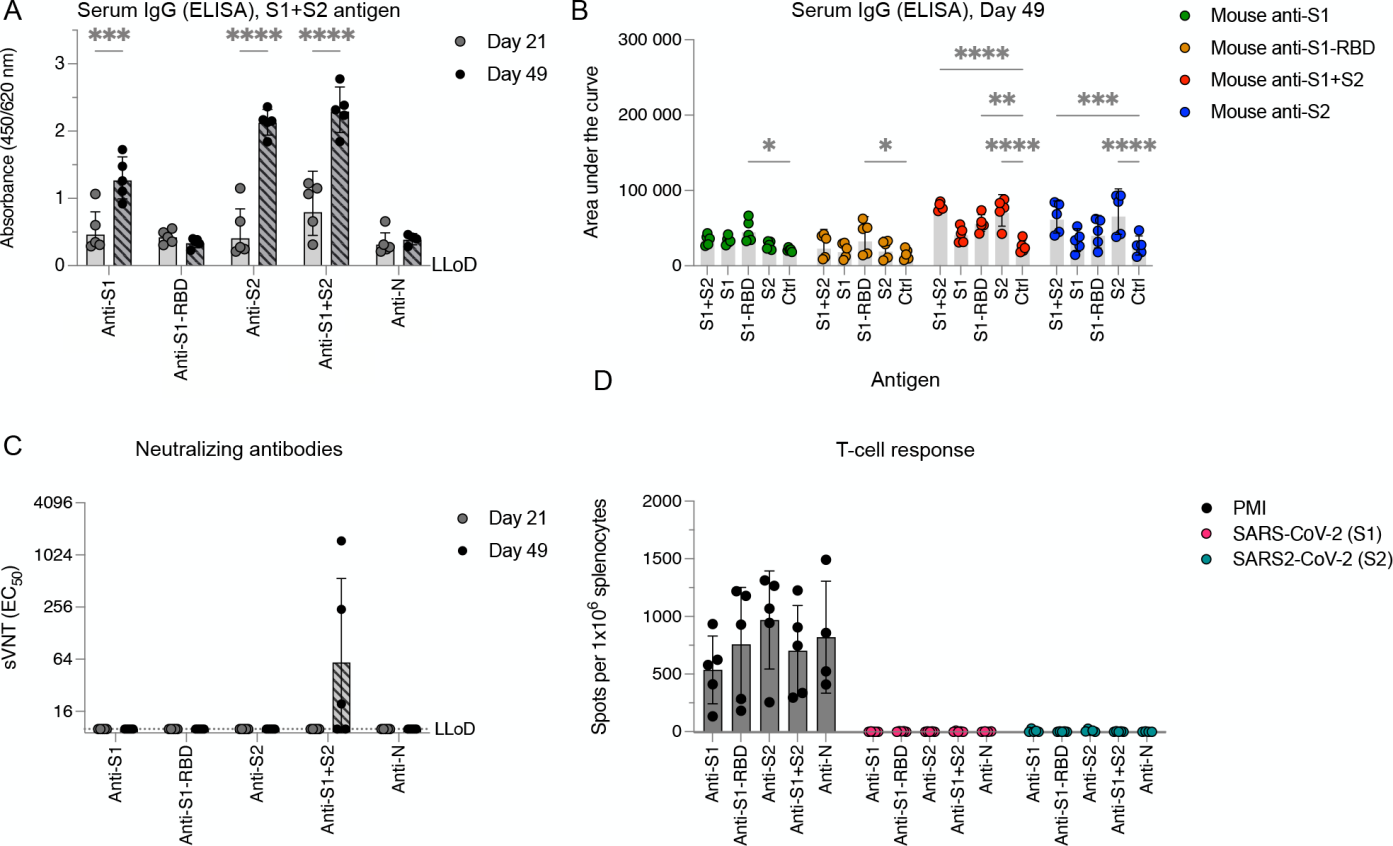
Poor pseudovirus-neutralizing antibody production and absence of T-cell responses to full-length SARS-CoV-2 spike ectodomain protein vaccination in IFNAR^−/−^-CD46Ge mice. (**A**) IFNAR^−/−^-CD46Ge mice were vaccinated intraperitoneally twice with 5 µg of alum-adjuvanted full-length spike ectodomain (S1 + S2), spike receptor-binding domain (S1-RBD), spike S1 domain (S1), spike S2 domain (S2), and nucleocapsid (N). Serum samples were collected on days 21 and 49, and the levels of spike ectodomain-binding IgG antibodies were quantified by enzyme-linked immunosorbent assay. (**B**) The binding IgG in serum was also quantified by ELISA for binding to homologous or heterologous antigens (*x*-axis). (**C**) Pseudovirus-neutralizing antibody titers were determined using pseudotyped viruses expressing the SARS-CoV-2 spike protein bearing the D614G amino acid change. (**D**) T-cell responses elicited against SARS-CoV-2 spike were determined by an enzyme-linked immunospot assay. The data are presented as IFN-γ-secreting cells or spot-forming cells per 1 × 10^6^ splenocytes, with values representing the geometric mean ± geometric standard deviation. Statistical significance was determined using two-way ANOVA with Bonferroni’s multiple comparison test (*, *P* < 0.05; **, *P* < 0.005; ***, *P* < 0.0004; ****, *P* < 0.0001), and only significant values are shown.

IFNAR^−/−^-CD46Ge mice were used as the preferred small-animal model for evaluating the antigenic properties of various SARS-CoV-2 vaccines. These mice were selected because they are considered the gold-standard model for the analysis of recombinant measles virus (rMeV)-based vaccine candidates (34). We initially sought to evaluate the full-length spike ectodomain (Wuhan-Hu-1 isolate, S1 + S2, amino acids 16 to 1213), the S1 domain (amino acids 16 to 685), the S2 domain (amino acids 686 to 1213), and the receptor-binding domain (amino acids 319 to 541) of SARS-CoV-2 as potential vaccine candidates. We immunized IFNAR^−/−^-CD46Ge mice twice at 3-week intervals with 5 µg of recombinant protein adjuvanted with aluminum hydroxide gel (alum). Alum was chosen because it is the most commonly used adjuvant in vaccines in humans. Serum samples were then collected on days 21 (before boost) and 49 to analyze the antibody response using enzyme-linked immunosorbent assay (ELISA). The results showed that the full-length spike ectodomain (S1 + S2) and the S2 subunit elicited the strongest IgG antibody response, while the S1-RBD and the S1 domains induced specific antibodies to the S1-RBD (Fig. 1A and B). The nAb response was measured using a lentiviral pseudotype assay (43) (Fig. S1), and neutralization activity was observed only in antisera generated in response to the full-length S1-S2 ectodomain, with low titers detected in only three out of five animals (Fig. 1C).

Finally, the T-cell response was assessed by analyzing splenocytes from immunized animals. Although a similar basic reactivity to nonspecific T-cell stimulation was observed, no reactivity was observed when splenocytes were stimulated *ex vivo* with SARS-CoV-2 spike peptides (Fig. 1D). Taken together, these data suggest that in the context of a spike protein-based vaccine adjuvanted with alum, only the complete SARS-CoV-2 spike protein could stimulate the immune system and exclusively activate the humoral arm of the immune response, displaying limited neutralizing activity.

### Multimerization of the SARS-CoV-2 spike protein enhances the pseudovirus-neutralizing antibody response

**Fig 2:**
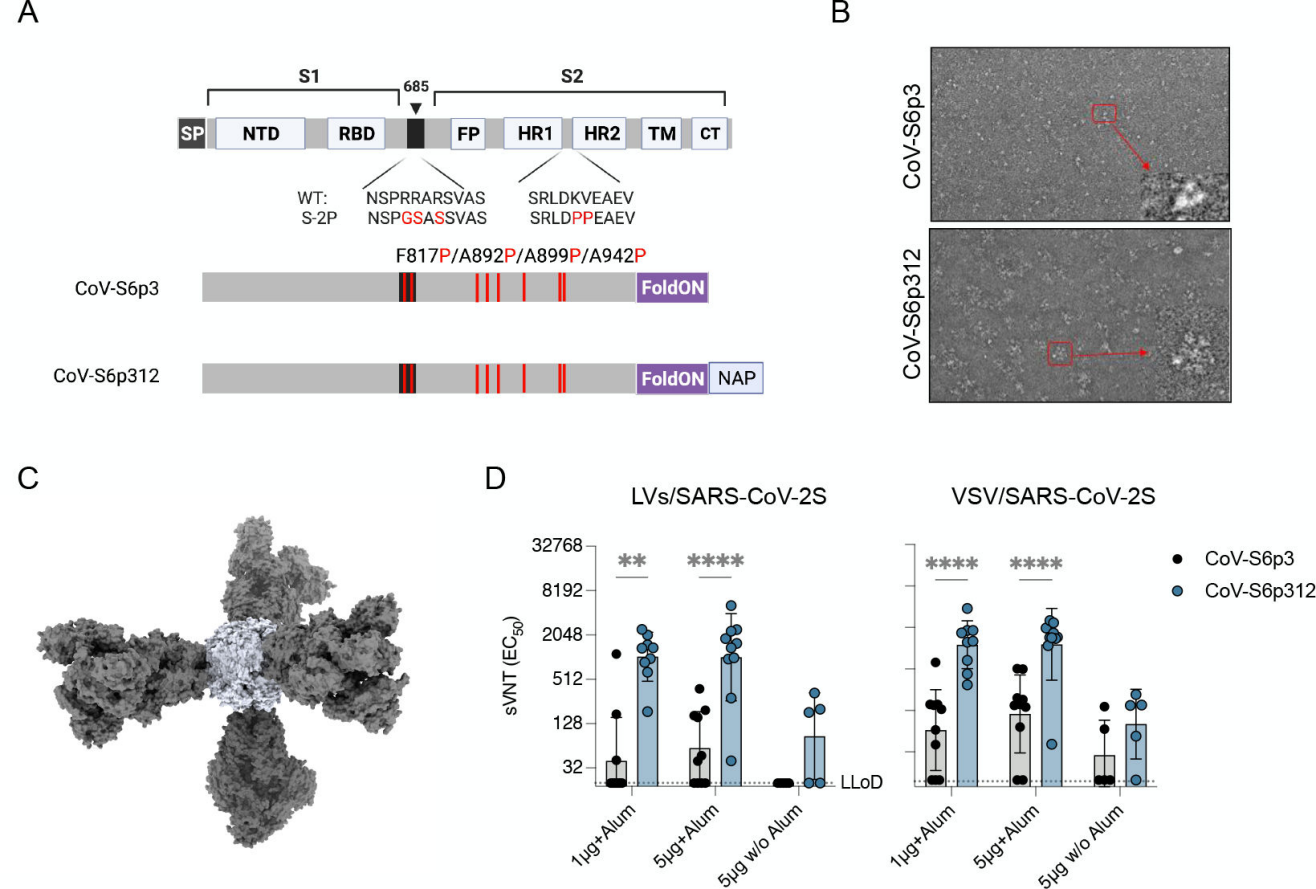
Multimerization of SARS-CoV-2 spike enhances neutralizing antibody responses. (**A**) The full-length SARS-CoV-2 spike and engineered full-length ectodomain spikes are illustrated in a schematic. Some of the structural domains shown include the cleavable signal peptide (SP), N-terminal domain (NTD), receptor-binding domain, S2 cleavage site (685, black), fusion peptide (FP), heptad repeats 1 and 2 (HR1 and HR2), transmembrane domain (TM) and cytoplasmic tail (CT). The native furin cleavage site was altered (RRAR→GSAS) to inhibit proteolytic cleavage, and six prolines, indicated in red text, were introduced to further increase stability. The C-terminal domain of the T4 fibritin (foldON, purple) was placed at the C-terminus of the spike, and the *H. pylori* neutrophil-activating protein (blue) was preceded by a GlySer linker. (**B**) Representative electron micrographs of negatively stained SARS-CoV-2S6p3 and SARS-CoV-2S6p312 proteins. The inset shows a close-up view. (**C**) Molecular surface representation of the SARS-CoV-2S6p312 protein. The structural model shows the dodecameric NAP protein [Protein Data Bank (PDB) ID: 1JI4, light blue] displaying four copies of SARS-CoV-2 HexaPro S (PDB ID: 6XKL, gray). (**D**) Mice were vaccinated once with either 1 or 5 µg of alum-adjuvanted proteins, and neutralizing antibodies in serum samples collected 21 days post-vaccination were quantified using LV-SARS-CoV-2 pseudoviruses (left panel) and VSV-SARS-CoV-2-S pseudoviruses (right panel). Black dots represent individual mice, and bars and error bars depict the geometric mean ± geometric standard deviation. Statistical analysis among groups was calculated by two-way ANOVA with Bonferroni’s post-test (***, *P* = 0.0001; ****, *P* < 0.0001).

Virtually, all human vaccines rely on the generation of nAbs for their effectiveness. Previous studies on other type I fusion glycoproteins have shown that nAbs primarily target metastable quaternary epitopes rather than monomeric forms (44–46). Consequently, we postulated that the observed disproportionally high ratio of binding antibodies to nAbs when a soluble purified protein was used for immunization could be attributed to the lack of quaternary assembly of the prefusion trimer. This would suggest that nAbs recognize a quaternary spike epitope in the metastable prefusion conformation, as observed in other type I fusion glycoproteins (10, 47–49). To begin to test our hypothesis, we incorporated a self-trimerizing T4 fibrin motif (foldON) (50) into the full-length spike ectodomain in conjugation with a mutated furin cleavage site in the spike and the previously reported stabilizing six proline substitutions in the spike (HexaPro, S-6p) (51), which disfavor the formation of an extended coiled coil (12). Additionally, we produced a genetic fusion at the C-terminus of the SARS-CoV-2 spike protein with *H. pylori* NAP (Fig. 2A). NAP is a 27-nm-wide dodecameric protein with four threefold axes (52), a feature that can enable the multivalent display of immunogens on the exterior surface. Next, both trimeric and full-length spike ectodomain (S1 + S2, herein termed CoV-S6p3) and CoV-S6p3-NAP (herein termed CoV-S6p312) were recombinantly expressed using mammalian cells to ensure the proper folding and glycosylation pattern of the proteins. SDS-PAGE analysis followed by Coomassie blue staining of purified CoV-S6p3 and CoV-S6p312 revealed apparent molecular weights of 180 and 210 kDa, respectively, under reducing conditions, which suggested the correct genetic fusion of NAP (Fig. S2A). This analysis also revealed that the preparations were of high purity. Further native gel electrophoresis demonstrated that both SARS-CoV-2 spike constructs preferentially assembled as mature trimers, as expected from a correctly fused foldON domain (three ~270 kDa units, Fig. S2B). The purity and homogeneity of the recombinant proteins were also verified by negative transmission electron microscopy (negative-TEM, Fig. 2B). We found a higher degree of protein aggregation after NAP conjugation, consistent with the nanoparticle display (Fig. 2C).

Finally, we compared the immunogenicity of these spike proteins by vaccinating 5–10 IFNAR^−/−^-CD46Ge mice with alum-adjuvanted formulations containing 1 or 5 µg of SARS-CoV-2 spike protein or with 5 µg of SARS-CoV-2 spike protein without alum. Serum samples were then collected at week 3 to measure the levels of pseudovirus nAbs. Mirroring the data presented above, not all the animals vaccinated with a prefusion, trimeric SARS-CoV-2 (CoV-S6p3) produced nAbs despite the use of the adjuvant (3/10 for the 1-µg dose and 6/10 for the 5-µg dose) and those that did exhibited low geometric mean titers (GMTs), i.e., 199 and 123. In contrast, all animals vaccinated with a homologous but covalently linked NAP-tagged SARS-CoV-2 spike (CoV-2S6p312) exhibited seroconversion when alum was used as an adjuvant and GMTs that were approximately fivefold higher, i.e., 1,038 and 1,021 for the 1- and 5-µg doses, respectively (Fig. 2D, left panel). Neutralization titers, when measured using VSV-SARS-CoV-2-S pseudoviruses, produced similar results (Fig. 2D, right panel). Nonetheless, the latter exhibited heightened sensitivity, especially at lower adjuvant concentrations. These data strongly suggest that the conformation of pertinent B-cell epitopes is likely to be maintained in both the metastable prefusion and stable postfusion products. We conclude from this experiment that the recombinant SARS-CoV-2 spike protein demonstrates low immunogenicity, but when displayed multivalently on a self-assembling nanoparticle scaffold, its immunogenicity is significantly augmented.

### Measles virus-based SARS-CoV-2 candidates express spike proteins with variations in oligomerization status

Considering that the full-length spike ectodomain could elicit only a modest nAb response in IFNAR^−/−^-CD46Ge mice, our aim shifted toward generating vaccine candidates for SARS-CoV-2, which are based on the resurfaced Moraten virus (MeV-MR) (42). Our decision to use MeV-MR was motivated by recent research, indicating decreased immunogenicity of a measles-vectored SARS-CoV-2 vaccine candidate in individuals who are seropositive for measles (41). Prior to the initiation of this study, we noticed that the immune response induced by the MeV-MR vector surpassed that induced by the MeV Moraten vaccine vector in the presence of anti-measles antibodies (Fig. S3). Hence, a panel of rMeV-MR variants containing the spike gene in unmodified or altered formats between the MeV-P- and MeV-M-coding sequences of MeV-MR was cloned (42). Among the modifications of the spike that were generated, we replaced the native signal sequence with the murine IgGκ leader sequence, followed by a hemagglutinin (HA) tag. This signal peptide (SP) has been shown to significantly boost the immunogenicity of an adenoviral-vectored vaccine platform (53). Additionally, we included two sets of prefusion-stabilized forms of spike protein, the S-2P construct (54) and the S-6P (14), for comparison. At the time of conducting this study, it was unclear which prefusion spike conformation would be more effective in inducing an immune response. Finally, we evaluated the product of the genetic fusion of NAP, with or without the presence of the foldON domain.

**Fig 3:**
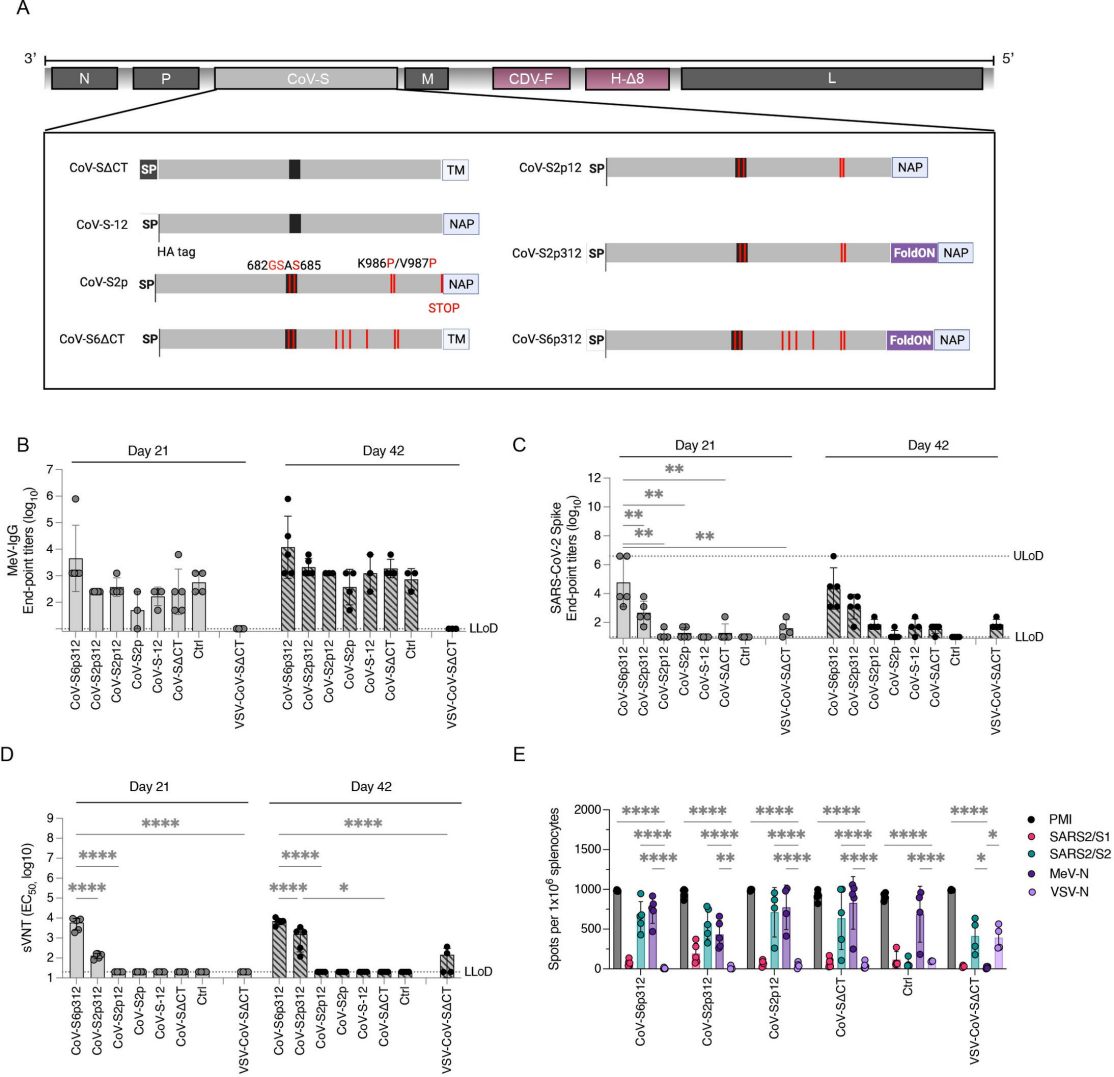
Trimerization and stabilization of SARS-CoV-2 spike protein constructs enhance humoral antibody and T-cell responses. (**A**) Schematic of the MeV-MR vector and various SARS-CoV-2 spike-based constructs inserted as an additional transcript unit, showing modifications to the transmembrane (TM) and cytoplasmic tail regions and substitution of the spike signal peptide with a murine IgG kappa leader sequence plus an HA tag. The *H. pylori* NAP was genetically fused at the C-terminus of the spike protein, optionally incorporating a stop termination codon or a foldON trimerization domain. (**B and C**) IgG-binding responses in IFNAR^−/−^-CD46Ge mice vaccinated with different recombinant viruses, as measured by ELISA for binding to (**B**) MeV-bulk antigen and (**C**) the spike ectodomain. (**D**) Pseudovirus-neutralizing antibody responses in mice vaccinated once or twice with the various recombinant viruses, as determined by pseudotyped lentiviruses expressing the SARS-CoV-2 spike D614G construct. (**E**) IFN-γ enzyme-linked immunospot assay for T-cell responses, showing the number of spot-forming cells per 1 × 106 splenocytes isolated from mice vaccinated twice and stimulated *ex vivo* with phorbol myristate acetate/ionomycin or antigen-specific peptides. Values are represented as the geometric mean ± geometric standard deviation, with each data point representing an individual mouse. Statistical significance was determined using one-way or two-way ANOVA with Bonferroni’s multiple comparison test (*, *P* < 0.05; **, *P* < 0.01; ***, *P* < 0.001; ****, *P* < 0.0001).

Altogether, we designed seven different constructs (outlined in Fig. 3A): (i) wild-type leader sequence with deletion of the S cytoplasmic tail (CoV-SΔCT); (ii) the CoV-SΔCT protein with an altered furin cleavage site and six stabilizing proline substitutions (CoV-S6ΔCT); (iii) the CoV-ΔCT protein with a murine IgGκ leader sequence and deletion of the spike transmembrane region, which represents the soluble ectodomain, fused to NAP (CoV-S-12); (iv) the CoV-S-12 protein with a modified furin cleavage site, two stabilizing proline substitutions, and a STOP codon prior to NAP (CoV-S2p); (v) the CoV-S2p protein without a STOP codon prior to NAP (CoV-S2p12); (vi) the CoV-S2p12 protein with a foldON trimerization motif between the prefusion spike and NAP (CoV-S2p312); and (vii) the CoV-S2p312 protein with four additional proline substitutions (CoV-S6p312).

**Fig 4:**
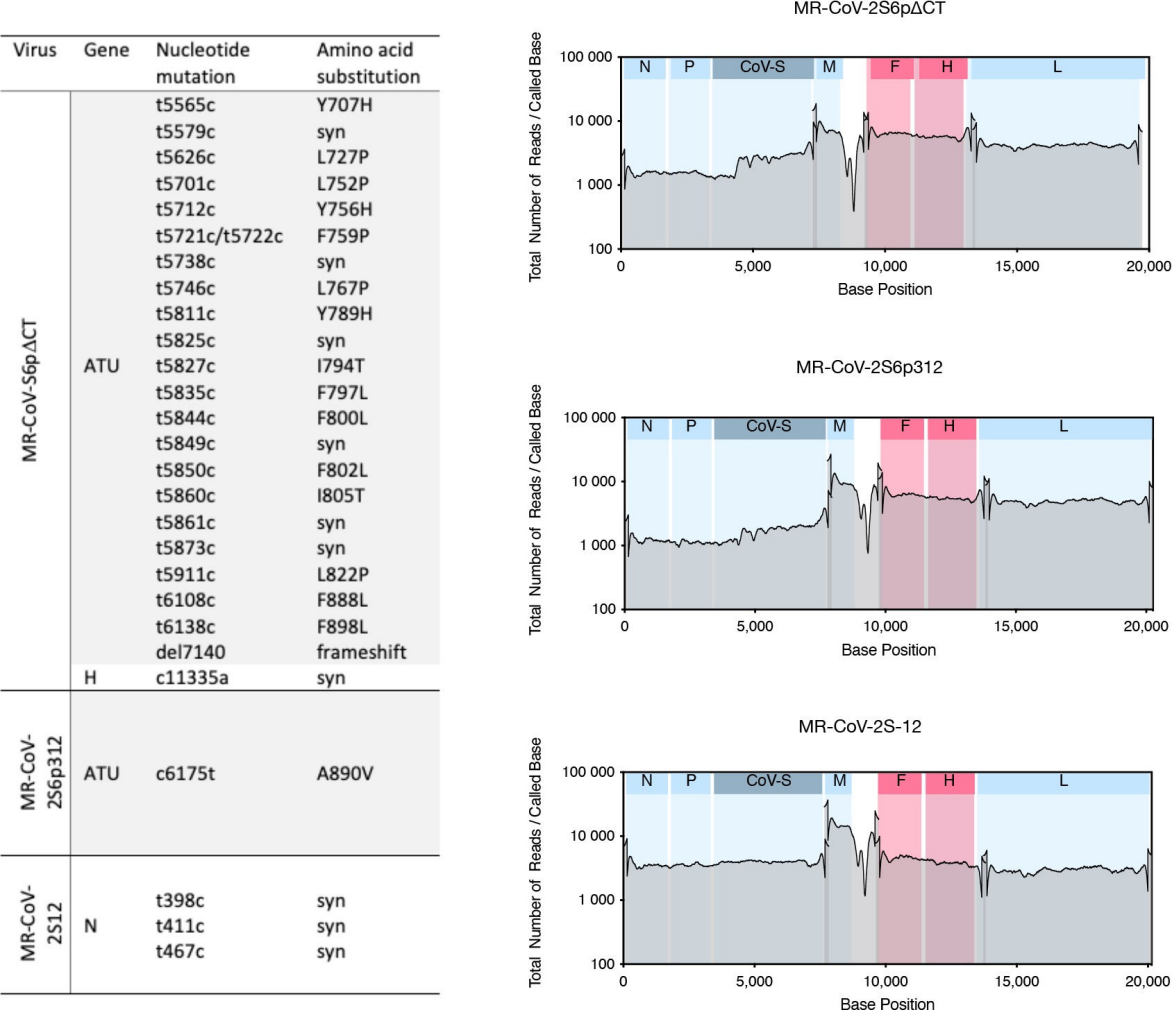
Sequence divergence of rescued viruses from cloned cDNA. The rescued viruses from cloned cDNA, in which nucleotide mutations were identified. The genome coverage and allelic frequency were determined by next-generation sequencing. Blue areas represent viral-coding sequences, while white areas indicate intergenic regions and untranscribed terminal regions of the genome.

The rMeVs were propagated in Vero cells to produce virus stocks, and the virus integrity was assessed by full-genome next-generation sequencing (NGS). The results showed that the coding sequences of MeV were identical, but some amino acid changes were noted in the spike region of some of the rMeVs. One of the vaccine candidates, MR-CoV-S6ΔCT, was abandoned because it showed 15 nonengineered amino acid changes and early termination due to a single-point mutation (Fig. 4).

The expression of the spike protein was then analyzed using western blot analysis of Vero cells infected with the rMeVs. The results showed that when the spike protein contained prefusion-stabilizing amino acid changes (S2p or S6p) and lacked the C-terminal NAP, it was primarily secreted into the culture medium in a soluble form (Fig. S4). In contrast, when NAP was added to the C-terminus of the spike protein, the spike protein was detected in both the culture medium and the cell pellet. Additionally, in cells that were infected with MR-CoV-SΔCT, both uncleaved S precursor and dissociated S2 fragments were found.

Overall, these results indicate that rMeV can effectively express a range of different spike protein constructs with varying oligomerization states and epitope accessibility.

### Artificial trimerization of the spike protein is critical for the immunogenicity of MeV-based SARS-CoV-2 vaccine candidates

We next evaluated the immunogenicity of the different vaccine candidates. To this end, 1 × 10^5^ plaque-forming units (pfu) of the various viruses were used to vaccinate 8- to 12-week-old IFNAR^−/−^-CD46Ge mice on days 0 and 21. Serum samples were then collected on days 21 (before boost) and 42 to assess the presence of S- and MeV-specific IgG antibodies by ELISA. As a control for vaccine immunogenicity, we utilized an isogenic MeV-MR encoding for Chikungunya virus (CHIKV) structural proteins (an irrelevant antigen) or a VSV-G protein-pseudotyped VSV encoding SARS-CoV-2 spike (VSV-CoV-SΔCT) (55).

The animals vaccinated with the different rMeV constructs showed seroconversion to MeV antigens 3 weeks after the initial vaccination, as demonstrated by the detection of MeV-specific IgG antibodies in their sera. These antibody levels increased by more than one log after a second dose, indicating the presence of vaccine-induced responses in all animals (Fig. 3B). Although MeV-specific IgG was detected in animals that received MeV-MR, seroconversion to SARS-CoV-2 was not observed in all groups, even after two doses. On the other hand, all animals vaccinated once with rMeV expressing a trimeric and stabilized SARS-CoV-2 spike (CoV-S2p312 or CoV-S6p312) and all animals vaccinated twice with rMeV-MR-CoV-S2p12 and VSV-CoV-SΔCT had detectable levels of SARS-CoV-2 spike-specific IgG antibodies in their serum (Fig. 3C).

The neutralizing activity of the antibodies was measured using SARS-CoV-2 spike-pseudotyped lentiviruses. Only mice vaccinated with stabilized, trimeric forms of the SARS-CoV-2 spike protein (CoV-S2p312 and CoV-S6p312) displayed significant levels of nAbs (Fig. 3D). Single doses of MR-CoV-S2p312- and CoV-S6p312-elicited nAbs, while two doses of VSV-CoV-SΔCT, were required to generate a noticeable response. Among the mice vaccinated with stabilized and trimeric forms of the spike proteins, those vaccinated with a single dose of MR-CoV-S6p312 displayed greater pseudovirus-neutralizing activity than those vaccinated with MR-CoV-S2p312. Additional experiments revealed that increasing the virus inoculum for the vaccination studies led to an increase in binding antibodies to SARS-CoV-2 spike. Nevertheless, pseudovirus neutralization activity remained largely unaltered (Fig. S5).

Furthermore, we assessed cell-mediated immunity by conducting an enzyme-linked immunospot (ELISpot) analysis on day 42. All mice that received rMeV-based vaccine candidates exhibited a robust response of IFN-γ-producing T cells (Fig. 3E). This response was observed even in animals that failed to mount a SARS-CoV-2-specific IgG response. The splenocytes obtained from these vaccinated animals exhibited reactivity to SARS-CoV-2 peptides. Notably, the reactivity was significant when the splenocytes were stimulated with a pool of SARS-CoV-2 spike spanning the S2 subunit (aa 633 to 1258). Our findings suggest that the intrinsic ability of SARS-CoV-2 spike protein to stimulate B-cell responses is weak. However, artificial trimerization and the inclusion of prefusion-stabilizing mutations are crucial in inducing SARS-CoV-2 spike IgG antibody production and intensifying nAb responses.

### Immunity elicited by MeV/SARS-CoV-2 is Th1 polarized

**Fig 5:**
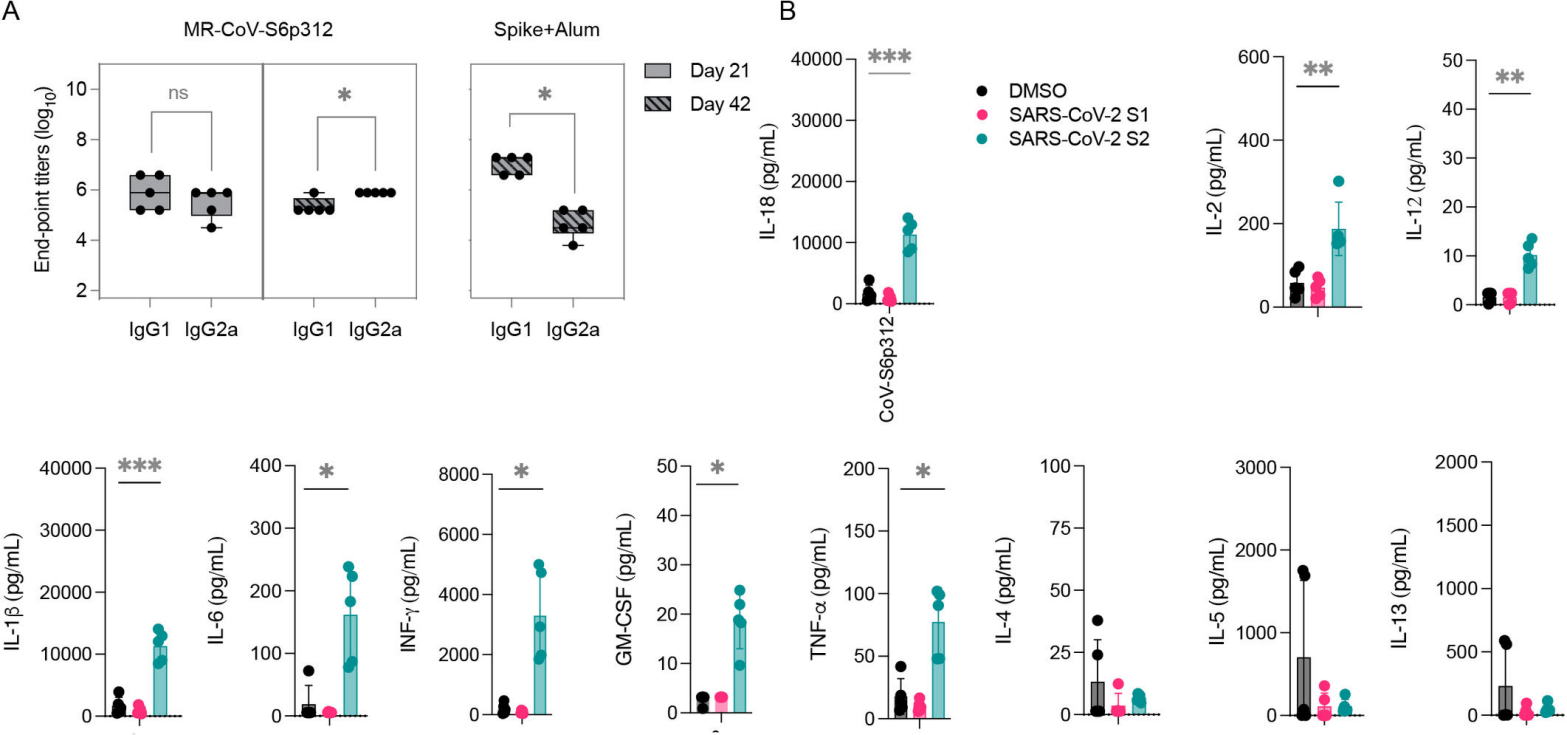
MR-CoV-S6p312 induces a Th1-biased immune response in IFNAR^−/−^-CD46Ge mice. (**A**) IFNAR^−/−^-isotype analysis of anti-SARS-CoV-2 spike antibodies in the serum of vaccinated mice, with ELISA measuring IgG1 and IgG2a antibody binding to SARS-CoV-2 spike. A control group of mice was vaccinated with purified SARS-CoV-2 spike adjuvanted with alum to compare the Th2-biased humoral response. (**B**) Cytokine production by splenocytes of vaccinated mice was analyzed using multiplex cytokine analysis after stimulation with SARS-CoV-2 peptides. Each dot represents an individual animal, and horizontal bars with error bars represent the mean ± SD. Statistical significance was determined using two-way ANOVA with Dunnett’s multiple comparison test (*, *P* < 0.05; **, *P* < 0.003; ***, *P* < 0.0003).

Vaccine-associated enhanced respiratory pathology after SARS-CoV-2 infection has been linked to a Th2-biased immune response (56, 57). To evaluate T-cell polarization, we next measured the levels of two IgG subclasses of SARS-CoV-2 spike-specific antibodies by ELISA, which served as an indication of T-cell polarization (58). As a control for a Th2-skewed response, we used serum from mice immunized twice with alum-adjuvanted SARS-CoV-2 spike protein (38). In these mice, we observed a significant difference (*P* < 0.05) in the levels of IgG1 and IgG2a subclasses, with IgG1 levels being significantly higher than IgG2a levels (Fig. 5A, right panel). In contrast, mice vaccinated with MR-CoV-S6p312 produced comparable IgG1 and IgG2a titers after one dose. After two doses, a statistically significant predominance of IgG2a was observed, indicative of a Th1-skewed response (Fig. 5A, left panel). We further confirmed the Th1/Th2 balance by analyzing the cytokine profile of splenocytes stimulated with a SARS-CoV-2 spike peptide pool. Specifically, splenocytes obtained from vaccinated animals were treated with DMSO or with the SARS-CoV-2 spike peptide pool, and cytokine secretion in the cell culture supernatant was quantified with a ProcartaPlex multiplex panel. The results showed strong Th1 polarization based on the production of IL-1β, IL-2, IL-12, TNF-α, and IFN-γ (Fig. 5B). No Th2-associated cytokines (IL-4, IL-5, and IL-13) were detected. Collectively, the assessments of both humoral and cellular responses revealed a desirable Th1-biased immune response elicited by MR-CoV-S6p312.

### MeV/SARS-CoV-2 vaccine based on the historical spike protein elicited low neutralizing antibodies against some SARS-CoV-2 variants

**Fig 6:**
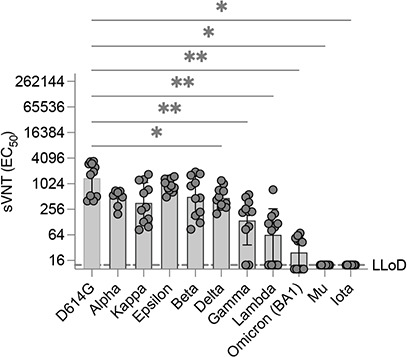
Neutralizing antibodies elicited by MR-CoV-S6p312 are susceptible to amino acid substitutions present in SARS-CoV-2 variants. The sensitivity of antibodies elicited by MR-CoV-S6p312 to SARS-CoV-2 variants was evaluated by measuring neutralizing activity using serum samples from animals vaccinated once with MR-CoV-S6p312. Pseudoviruses bearing the SARS-CoV-2 spike from different variants were used to determine neutralizing antibody responses. Individual mouse sera are represented by black dots, and bars and error bars depict the geometric mean ± geometric standard deviation, respectively. Statistical analysis was performed using one-way ANOVA with Bonferroni’s post-test (*, *P* < 0.05; **, *P* < 0.01).

We next proceeded to assess whether this favorable Th1-type immune response triggered by vaccination with MeV could neutralize the SARS-CoV-2 variants that have predominantly replaced the original SARS-CoV-2 strain used for our vaccine design. As of January 2022, the World Health Organization had defined five VOCs, namely, Alpha (B.1.17), Beta (B1.351), Gamma (P1), Delta (B.1.617.2), and Omicron (B.1.1.529), as well as five variants of interest [Iota (B.1.526), Kappa (B.1.617.1), Lambda (C.37), Mu (B.1.621), and Epsilon (B.1.427/B.1.429)]. Among these, the Omicron variant (previously known as B1.1.529) is of great concern because it contains 34 amino acid substitutions in the spike protein compared to the 8–12 substitutions seen in previous VOCs. This has led to partial or complete escape of the humoral (59, 60) but not the T-cell responses elicited by vaccination using different platforms (59–62). Our primary goal was to evaluate the effectiveness of the MR-CoV-S6p312 vaccine candidate in generating nAbs against different SARS-CoV variants. For this purpose, we performed antibody neutralization assays with pseudoviruses that expressed the Omicron BA.1 variant SARS-CoV spike, which contained several lineage-defining amino acid changes. We also generated additional pseudoviruses that harbored the spike proteins of various other variants. We found that sera from mice vaccinated with a single vaccine dose of MR-CoV-S6p312 had similar neutralizing effects (*P* > 0.05) on pseudoviruses that harbored spikes from Alpha, Kappa, Epsilon, and Beta. However, a partial or complete loss of neutralization was observed for pseudoviruses that harbored spikes from Delta, Gamma, Lambda, Omicron BA.1, Mu, and Iota (Fig. 6). Hence, we observed that some variants were resistant to neutralization by antibodies generated to a single dose of our measles-vectored COVID-19 vaccine candidate. This implies that a single dose of the vaccine may not be sufficient to provide protection against certain SARS-CoV-2 variants.

### An Omicron-matched MeV/SARS-CoV-2 vaccine candidate restores nAb titers against historical and BA.1 variants

To address the inadequate nAb response to the Omicron lineage variant BA.1 following a single vaccination with MR-CoV-S6p312, we sought to evaluate (i) whether the original (wt) MR-CoV-S6p312 vaccine candidate generated a limited immune response and (ii) whether a homologous wt MR-CoV-S6p312 or a heterologous Omicron BA.1-matched MR-CoV-S6p312 vaccine candidate could augment the neutralizing responses to the Omicron variant. Additionally, we aimed to determine whether there was a difference in the immune response between homologous and heterologous boosting.

**Fig 7:**
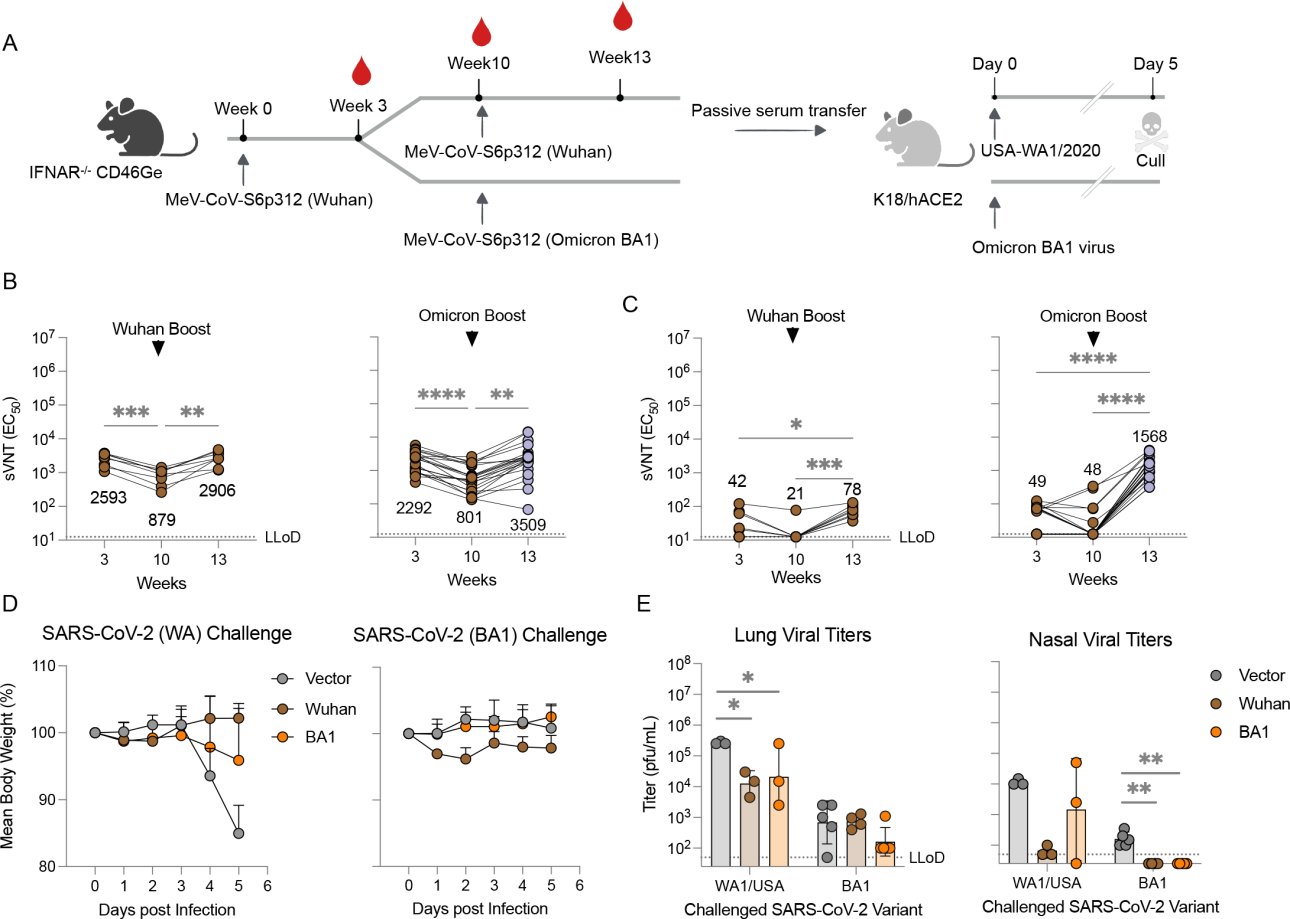
Boosting with an Omicron BA.1-matched MeV/COVID-19 vaccine candidate enhances neutralizing activity and confers protection in K18-hACE2 mice. (**A–C**) IFNAR^−/−^-CD46Ge mice were vaccinated with D614G-based MeV/SARS-CoV-S6p312 at week 10 and boosted with MR-CoV-S6p312 based on the same ancestral D614G spike or an Omicron BA.1-based spike at week 10. Serum samples were collected at weeks 3, 10, and 13 and analyzed for pseudovirus-neutralizing antibodies using a VSV/SARS-CoV-2 pseudovirus based on the Wuhan spike (**B**) or Omicron BA-1 spike (**C**). Individual mouse sera are represented by dots. Statistical analysis among time points was calculated by one-way ANOVA with Bonferroni’s post-test (*, *P* < 0.05; **, *P* < 0.006; ***, *P* < 0.0007; ****, *P* < 0.0001). (**D**) Passive immunization of K18-hACE2 mice with serum samples from previously vaccinated IFNAR^−/−^-CD46Ge animals was performed to assess protection against body weight loss in mice challenged with authentic SARS-CoV-2 viruses. Two hours later, K18-hACE2 mice were challenged intranasally with WA1/2020 (Wuhan-like strain) or hCoV-19/USA/NY-MSHSPSP-PV44476/2021 (a BA.1 strain) and monitored for body weight loss. Dots depict the mean ± standard deviation. (**E**) Virus burden in homogenates from lung and nasal turbinates at 5 days post-challenge with WA1/2020 or Omicron BA.1 virus was assessed by plaque assay. Individual animals are represented by dots, while the geometric mean ± geometric standard deviation is depicted by horizontal bars and error bars. Statistical significance between groups was calculated by one-way ANOVA with Dunnett’s post-test (*, *P* < 0.05; **, *P* < 0.01).

To address these questions, two groups of 15- to 17-week-old IFNAR^−/−^-CD46Ge mice were vaccinated sequentially at weeks 0 and 10, with either two doses of wt vaccine or a combination of one dose of wt and another dose of the BA.1-matched MR-CoV-S6p312 vaccine candidate (Fig. 7A). We used a 10-week period between vaccination doses to ensure the presence of affinity-mature, class-switched memory B cells and long-lived plasma cells. Blood samples were collected at peak levels (week 3) (63), prior to boosting (week 10), and 3 weeks thereafter (postboosting), and the presence of VSV-SARS-CoV-2-S pseudovirus-nAbs was measured. The results showed significant lower neutralization activity against the Wuhan strain in the serum samples from week 3 compared to those from week 10 (threefold, *P* < 0.0005) in mice vaccinated with the wt vaccine (Fig. 7B). Consistent with our previous results, Omicron nAbs were low or absent in these animals (Fig. 7C). A subsequent booster shot of the wt vaccine significantly increased the levels of Wuhan strain nAbs (*P* < 0.0005), reaching levels comparable to those in week 3 after the first dose (Fig. 7B, left panel). Although Omicron strain nAbs were detected in all the animals, the GMT of neutralization activity was low, i.e., (1/dilution) ± SEM of (72.8 ± 11.0) (Fig. 7C, left panel). However, a booster shot based on the Omicron variant not only increased the antibody titers against the Omicron variant but also restored the antibody titers against Wuhan strain pseudoviruses (Fig. 7B and C, right panels). The neutralization titers against the Wuhan strain pseudovirus were equivalent (*P* > 0.05) between animals receiving either the wt or Omicron-based boosters. Together, these findings strongly suggest that Omicron could be considered a SARS-CoV-2 serotype (64) and, therefore, that a booster based on the Omicron variant may be better suited to restore nAb titers against both the homotypic virus and historical SARS-CoV-2 variants.

### Vaccine-elicited antibodies from historical and Omicron-matched MeV/SARS-CoV-2 vaccine candidates protect against SARS-CoV-2 challenge

To evaluate the effectiveness of our antibodies *in vivo* against SARS-CoV-2, we conducted a study to assess the protective efficacy of homologous and heterologous boosts through passive antibody transfer. We pooled sera from IFNAR^−/−^-CD46Ge mice that received a booster dose of either wt or Omicron-based MR-CoV-S6p312 and used these sera for instillation into K18-hACE mice, which express hACE2 under the control of the epithelial cytokeratin promoter (65). We also used serum from IFNAR^−/−^-CD46Ge mice that received two doses of a MeV-MR empty vector as a control. The animals were then challenged intranasally with either 10^4^ pfu of USA-WA1/2020 SARS-CoV-2 (Wuhan-like) or Omicron BA.1 virus. We monitored mice for clinical signs of disease, including daily weight changes, and on day 5 post-infection, we euthanized the mice and collected their lung tissue and nasal turbinates to determine virus titers through plaque assay. This approach allowed us to fully evaluate the potential of the humoral response to protect against infection.

The mice challenged with USA-WA1/2020 showed no signs of weight loss when pretreated with vaccination serum. In contrast, the mice in the control group displayed a decline in weight starting from the fourth day post-infection (dpi) (Fig. 7D). Despite considerable viral replication in the lungs of passively immunized mice, both types of vaccination serum yielded similarly reduced lung viral titers (∼16-fold, Fig. 7E). The nasal viral titer was substantially reduced by 175-fold in the mice that received serum from the mice vaccinated with wt MR-CoV-S6p312 in comparison to the control group.

In mice that were challenged with the BA.1 virus, we did not observe any significant decrease in body weight loss, and the viral titers detected in the lung and nasal turbinates were ∼100-fold lower than those detected with the USA-WA1/2020 virus (Fig. 7E), as previously reported (66). Notably, all vaccinated mice had detectable SARS-CoV-2 in their lungs after challenge, but no infectious virus was present in their nasal turbinates. Mice vaccinated with the Omicron-based MR-CoV-2S6p312 vector showed approximately fourfold lower lung viral titers than those treated with the wt construct and control group. Thus, as shown in a previous study (22), protection against BA.1 was modestly improved in animals that received a BA1-based booster vaccine. We conclude from this experiment that *in vitro* antibody neutralization does not necessarily correlate with efficacy in a prophylactic setting.

### MR-CoV-S6p312 elicits SARS-CoV-2 spike-specific nAb responses in the presence of preexisting measles antibodies

**Fig 8:**
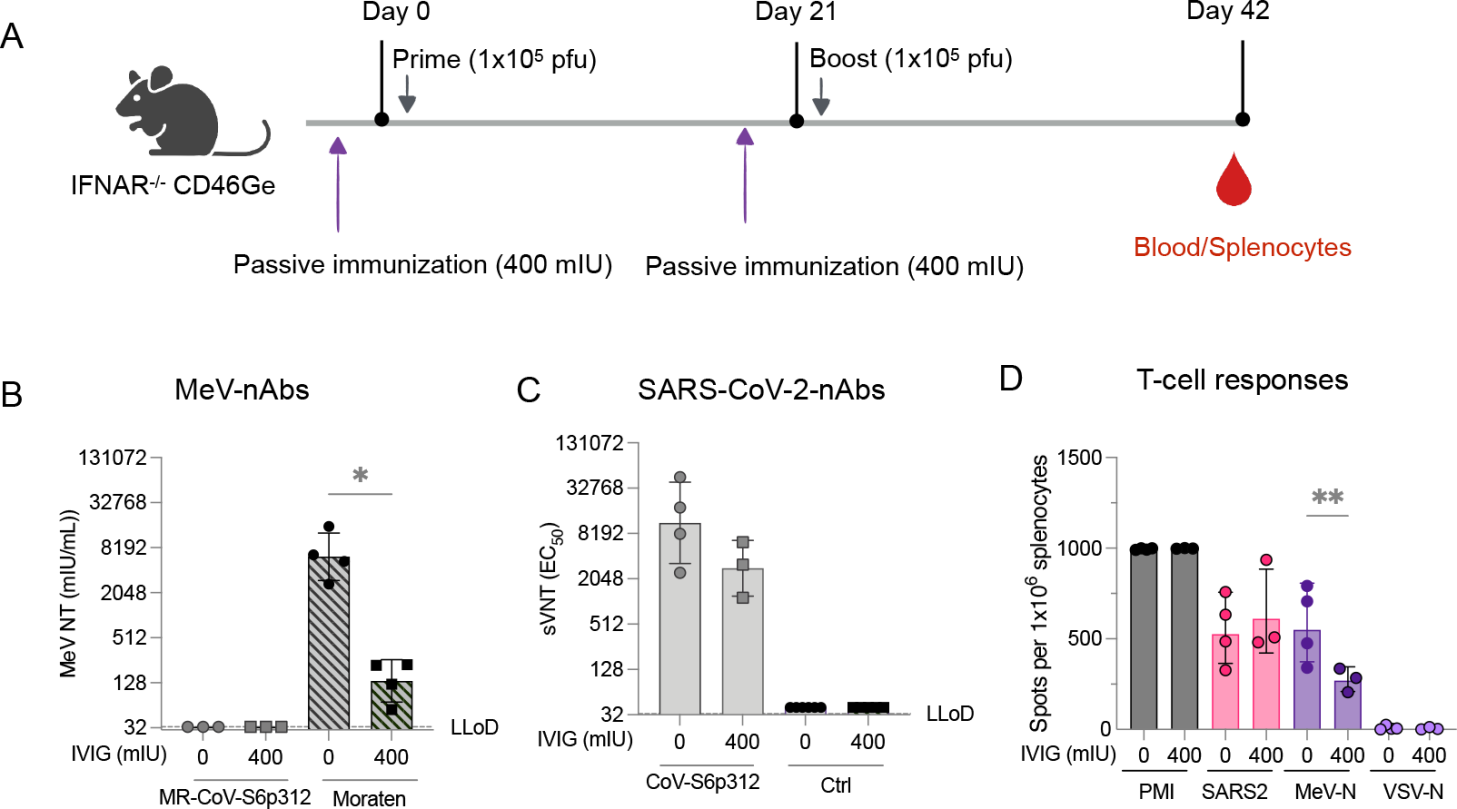
Preexisting anti-measles virus does not impact the immune response to SARS-CoV-2 spike elicited by MR-CoV-S6p312 vaccination. The effect of preexisting anti-measles virus immunity on the immune response to MR-CoV-S6p312 vaccination. (**A**) The experimental design involved passive immunization of IFNAR^−/−^-CD46Ge mice with anti-measles virus-neutralizing antibodies before each vaccination with MR-CoV-S6p312. Serum samples were collected 3 weeks after the second vaccination dose and analyzed for (**B**) measles virus-neutralizing antibodies and (**C**) SARS-CoV-2 spike pseudovirus-neutralizing antibodies. (**D**) Splenocytes were collected 3 weeks after the second vaccination, and ELISpot assays were performed. Individual mice are represented by dots, while the geometric mean ± geometric standard deviation is depicted by bars and error bars, respectively. Statistical analysis between groups was calculated by one-way ANOVA with Fisher’s LSD test (*, *P* < 0.05; **, *P* < 0.005).

A previously developed MeV/SARS-CoV-2 vaccine failed to elicit any nAb response in people with preexisting immunity to measles (40, 41). In our mouse model, we found that previous vaccination with MeV Moraten, but not MeV-MR, reduced the immune response to the SARS-CoV-2 spike from a Moraten-CoV-S6p312 vaccine candidate (Fig. S6). We postulated that our MeV-MR vector might be less vulnerable to this so-called “blunting effect” if nAbs against the MeV coat are responsible for attenuating the heterologous humoral immune response to the transgene. To begin to address whether the previously observed pseudovirus-neutralizing responses could be impacted by measles immunity, we vaccinated IFNAR^−/−^-CD46Ge mice in the presence or absence of MeV-specific IgG. To this end, 400 mIU of MeV nAbs was administered 3 hours prior to vaccination with either the MR-CoV-S6p312 or the MeV Moraten vaccine, which was used as a control. Three weeks later, after a second passive administration of MeV nAbs, the animals received a booster. We then assessed the nAb response against MeV (Moraten vaccine) or SARS-CoV-2 pseudoviruses, as well as the T-cell immunity (Fig. 8A). As expected, no MeV nAbs were detected in mice vaccinated with MR-CoV-S6p312 due to the divergence of the MeV coat (42). In contrast, naïve animals vaccinated with the homologous Moraten virus developed a mean MeV neutralization titer of 6,194 mIU/mL. However, when animals were passively immunized, the production of MeV nAbs was drastically reduced to 136 mIU/mL (Fig. 8B), indicating that we successfully modeled the impact of preexisting anti-MeV antibodies on the immunogenicity of the measles vaccine (67, 68).

Data were collected at the same time point to analyze the immune response against the SARS-CoV-2 spike protein generated in response to MR-CoV-S6p312. The levels of pseudovirus nAbs were found to be comparable between naïve animals and those with preexisting anti-MeV antibodies (Fig. 8C). As expected, no pseudovirus nAbs were detected after vaccination with the MeV Moraten vaccine. ELISpot assays performed 3 weeks after the second dose revealed no significant differences (*P* > 0.05) in the number of SARS-CoV-2 spike-specific IFNγ-producing cells in the animals vaccinated in the presence or absence of preexisting anti-MeV antibodies (Fig. 8D). However, a significant decrease in the number of MeV-N-specific IFN-γ-producing cells was observed. In conclusion, preexisting MeV nAbs had no negative impact on the immunogenicity of our MeV-MR-vectored vaccine since the resulting titers were comparable to those observed in naïve animals. Collectively, our results suggest that a MeV/SARS-CoV-2 vaccine candidate based on a remodeled MeV may serve as an efficient approach to induce nAb responses against SARS-CoV-2 in a measles-immune human population.

## DISCUSSION

In this study, our aim was to develop a remodeled live-attenuated measles vaccine that could generate strong nAb responses against the SARS-CoV-2 spike protein. Our findings show that antibodies produced by a measles-based COVID-19 vaccine candidate can offer protection against morbidity caused by SARS-CoV-2 infection in mice. Our work provides direct concrete evidence that antigen optimization of the SARS-CoV-2 spike protein significantly enhances nAb production. We based these conclusions on several pieces of evidence. First, we demonstrated that artificial trimerization of the SARS-CoV-2 spike protein is crucial to induce an effective humoral immune response against the spike protein by using measles-based COVID-19 vaccine candidates. Second, we showed that further multimerization of the SARS-CoV-2 spike protein through genetic fusion to the *H. pylori* NAP increases the magnitude of the nAb response. Third, we showed that boosting with an Omicron-based COVID-19 vaccine candidate restores neutralizing activity against both historically and contemporarily identified SARS-CoV-2 variants. Finally, we presented compelling evidence that preexisting anti-MeV antibodies do not impact the immunogenicity of MeV-based COVID-19 vaccine candidates containing epitope-modified H and F surface glycoproteins (MeV-MR). Altogether, our results strongly support the further development of MeV-MR-based vaccine candidates expressing the engineered SARS-CoV-2 spike protein to induce a protective immune response in individuals previously immunized with measles.

The Comirnaty (BioNTech/Pfizer) and SpikeVax (Moderna) COVID-19 vaccines have saved millions of lives owing to their unprecedented speed of development and high degree of efficacy (69). During the first year of the pandemic, the two vaccines provided >95% efficacy against symptomatic infection, but since then, there has been a marked decline in their ability to prevent infection, most likely due to the evolution of immune-evasive variants. Although booster shots can “restore” nAb responses and protection against variants (22, 70, 71), their use will not bring an end to this pandemic, rendering it imperative to develop next-generation vaccines. The live-attenuated MeV vaccine, which induces both humoral and cellular immune responses that can endure a lifetime, is also one of the safest human vaccines ever developed, with outstanding safety records in children <5 years old. These two features make the live-attenuated MeV vaccine an attractive platform for vectoring vaccines against other pathogens. Although whether the longevity of the protection against measles is applicable to other diseases remains unknown, the high seroprevalence of MeV in the human population limits these studies, as exemplified recently by a phase I/II clinical trial of a measles-vectored SARS-CoV-2 vaccine candidate (41).

Here, we used our recently described MeV-MR to counter the compromise of vector immunogenicity (42, 72). Our prior research demonstrated that, in contrast to the MeV vaccine strain, MeV-MR replicated *in vivo* in animals with passive immunity (42). To investigate how to improve the immunogenicity of a MeV-MR-based COVID-19 vaccine candidate, we specifically examined the antibody response elicited by various SARS-CoV-2 spike antigens in mice. We found that while both the S1 +S2 ectodomain and the S2 subunit were able to induce similar levels of IgG antibodies that bound to both antigens, only the S1 + S2 ectodomain elicited pseudovirus nAbs. These findings support the results of previous studies that have shown the limited immunogenicity of S2 in both mice and rabbits (73, 74). Our study found low levels of nAb responses to both the purified spike ectodomain and the full-length S construct of MeV that include the native transmembrane trimerization motif (75). Although the lack of a quaternary structure for the purified spike ectodomain may have contributed to suboptimal responses, two prior studies on MeV-based vaccine candidates expressing full-length spike also reported similarly low-to-absent nAb responses in cotton rats (39) and IFNAR^−/−^ KO CD46 mice (38), indicating that our findings are relevant to other vectors and species. While it is known that multimeric antigens generally elicit stronger immunogenicity than soluble antigens (76, 77), it was previously unclear whether a trimerization motif could enhance the immunogenicity of the full-length ectodomain of the SARS-CoV-2 S protein (39, 78, 79). To address this, we designed a trimeric prefusion SARS-CoV-2 S protein by incorporating the foldON trimerization motif and prefusion-stabilizing substitutions (14) (SARS-CoV-2S6p3), building on previous work on the prefusion conformation of the viral envelope and its antigenicity (10, 47, 80). Our results showed that a single injection of 5 µg of purified protein, adjuvanted with alum, remained inadequately immunogenic, as seroconversion rates ranged from 60% to 80%. However, subsequent multimerization by adding NAP (S6p312) significantly improved the immunogenicity of the spike protein.

We next studied the immunogenicity of different spike antigens in the context of MeV-vectored vaccine candidates. Although there were no significant differences in antibody titers as measured by ELISA, only constructs containing an artificial trimerization domain and NAP induced the production of pseudovirus nAbs. Notably, an S6P construct induced more uniformly high neutralizing titers than its S-2P counterpart when fused with NAP. Our findings suggest that although improved protein expression and stability do not always result in better immunogenicity, the use of a spike protein with six stabilizing proline substitutions (HexaPro construct) resulted in higher neutralizing antibody titers than the version with two prolines (81). This is consistent with a recent study by Lu et al. (82) published during the preparation of this manuscript. While an mRNA COVID-19 vaccine candidate based on the HexaPro variant developed by Sanofi Pasteur failed to elicit nAbs in mice and nonhuman primates, the latter construct lacked a trimerization domain (83). Therefore, an mRNA COVID-19 vaccine candidate based on the HexaPro variant fused to a trimerization peptide domain is likely to be a promising vaccine candidate. Furthermore, a clinical grade Newcastle disease virus/COVID-19 vaccine candidate has been developed based on the HexaPro variant, with the transmembrane domain and cytoplasmic tail of NDV (NDV-F) replacing those of the spike (84). However, the capacity of the NDV-F transmembrane region and cytoplasmic tail to trimerize the SARS-CoV-2 spike protein remains to be determined.

As evidenced with other vaccine candidates for COVID-19 (22, 70, 85), certain variants of SARS-CoV-2 partially or completely escaped the nAbs produced after a single vaccination with MR-CoV-S6p312. However, a second vaccination dose with either wt or BA.1-based MR-S-CoV-2S6p312 substantially increased the Wuhan-specific nAb titers to peak values after the initial vaccination. Notably, titers of nAbs against BA.1 were lower after a second dose of homologous MR-CoV-S6p312 than those observed following vaccination with the heterologous yet properly matched MR-CoV-S6p312 BA.1, which is consistent with previously published data (22, 86, 87). Protection against challenge with the BA.1 variant was not clearly correlated with the nAb titer, as observed in the limited number of K18-hACE mice used in this study. Although boosting with BA.1-based MR-S-CoV-2S6p312 reduced viral titers in the lower respiratory tract compared to the wild type, the difference was not statistically significant. This disparity might be attributed to the nonneutralizing Fc effector function of antibodies, which was not accounted for in this study. Alternatively, a larger animal experiment or pathological analysis of lung sections might produce further insights. Nonetheless, other studies have reported only a modest, if extant, protective effect against BA.1 challenge when the vaccine is matched to BA.1 (22, 70).

Viral titers in the nasal cavity displayed a more marked reduction than those detected in the lungs. These observations are in close agreement with findings from previous studies (88). The varying response between the two regions may be a consequence of the higher viral load in the lungs relative to the upper respiratory tract. Such a concentration gradient complicates the neutralization process, especially given the relatively low dose of passively administered antibodies. Moreover, the time interval between passive antibody administration and the ensuing viral challenge points to a prophylactic strategy for the antibodies’ delivery. Because of this timing, the antibodies might not achieve full equilibration by the point of viral encounter (89, 90). Consequently, the antibodies’ primary effector mechanisms predominantly arise during inflammatory responses. It is worth noting that viral replication in the upper respiratory tract occurs at a later stage; during this phase, antibodies have more effectively spread throughout the tract, augmenting their protective role.

It is interesting to note that there are differences in the immune response to different antigens depending on the vector platform used. For instance, while adenovirus 26 and modified vaccinia Ankara virus expressing the full-length, unmodified SARS-CoV-2 S protein have induced strong neutralizing responses (91, 92), we and others have observed a low-to-absent nAb response with MeVs expressing a similar construct (38, 39). Likewise, Mercado et al. (93) observed that an Ad26 expressing a secreted 2P-stabilized S antigen with the deletion of the S1/S2 furin cleavage site and a foldON trimerization motif replacing its TM and CT domains (S.dTM.PP) elicited nAb titers comparable to those produced using Ad26 expressing the unmodified full-length SARS-CoV-2 S protein (S and S.dCT). However, in the context of MeV vaccination, a homologous construct using the trimeric, secreted S-2P form was clearly superior (39). We did not pursue the generation of the MeV-based recombinant vaccine candidate that was reported by Lu et al. (39) (preS) due to the inadequate immunogenicity of the protein observed even when we used as a more stable HexaPro S variant (Fig. 2). Seroconversion was not observed in all of the animals. Additionally, Lu et al. did not report nAb titers in mice nor did they compare spike-binding antibodies with the membrane-embedded form. Furthermore, the binding antibody levels in mice were achieved with a high dose of rMeV, 10 times higher than what we used in this study. Therefore, the protective effect observed in the study by Lu et al. following SARS-CoV-2 challenge may have been produced solely by cellular immune memory, as previously demonstrated in the hamster model (94).

Frantz et al. (37) recently reported another MeV-based COVID-19 vaccine candidate that used a prefusion-stabilized full-length S (S-2P) membrane-anchored antigen (SF-S2-dER). The construct effectively protected different rodent models from SARS-CoV-2 challenge in the context of active vaccination. Although a direct comparison between MeV vectors expressing SF-S2-dER or preS has not been reported, both constructs have been studied in the context of Ad26 infection (93, 95). Based on the extrapolation of similar immunogenicity across platforms, we expect the MeV-based COVID-19 candidate presented in our study to elicit four- to sixfold more nAbs than the construct reported by Frantz et al. However, it is important to note that using a soluble spike over a membrane-anchored spike is antigen camouflage/decoy to prevent interference by preexisting antibodies. This attribute could be particularly important in the case of vectored vaccines as booster shots in people who were already infected or vaccinated against SARS-CoV-2. Additionally, it is necessary to consider the potential tropism expansion of the new vector after the incorporation of viral glycoproteins. These risks, along with the possibilities of vaccine-induced disease in certain populations and of cross-species transmission, make the regulatory process complicated and may negate the already established safety profile of the vector, as well as affect existing manufacturing processes.

To improve the antigenicity and durability of immune responses, several vaccines have required enhancements. Bacterial vaccines, such as the Hib vaccine or the pneumococcal vaccine, use protein conjugates to extend the period of protection against disease provided by the vaccines (96, 97). Self-assembling protein nanoparticles that can multivalently display viral antigens have proven to be an effective approach for augmenting the magnitude and breath of nAb responses (13). These multivalent vaccines can efficiently interact with antigen-presenting cells, which migrate to lymph nodes and amplify immune processing (98, 99). However, class I viral fusion proteins have not always been presented in the native trimeric form on nanoparticles (100–102). Our results show that adding an exogenous trimerization motif to the SARS-CoV-2 spike protein is critical for enhanced nAb responses when displayed on NAP. Recent studies have reported that the genetic fusion of ferritin to Ebola glycoproteins had no effect on the elicited antibody responses (103), perhaps due to the lack of a trimeric Ebola GP, similar to the results we have demonstrated for SARS-CoV-2. Hence, our study may provide insight into developing next-generation vaccine candidates based on class-I viral antigens.

In contrast to other nanoparticle vaccines for SARS-CoV-2 that require *in vitro* assembly and purification of the nanoparticle complex, our nanoparticle vaccine is unique in that all components are naturally sourced and encoded in the measles vector. This approach may simplify the manufacturing process and reduce costs. Additionally, the safety of our vaccine is supported by previous phase I clinical trials of recombinant NAP as a vaccine candidate for *H. pylori*, as well as an ongoing phase I clinical trial of an oncolytic measles virus expressing NAP to treat metastatic breast cancer patients (104, 105). Therefore, our measles vector-based nanoparticle vaccine is a promising and safe candidate for the prevention of SARS-CoV-2 infection.

Our study has several limitations that need to be addressed in future research (1). The sample size of K18-hACE2 mice was limited, and further experiments with larger cohorts are needed to validate and generalize our findings (2). We analyzed the immune response and protection against BA.1 Omicron, but currently, BA.4/5 are now the dominant Omicron-lineage viruses, and the FDA recommends that newer vaccines contain these latest Omicron variant sequences (106). We nonetheless do not expect our results to deviate significantly based on the premise that, in the context of breakthrough infection, prior BA.1 infection provides substantial protection against BA.5 (107, 108). Similarly, two bivalent mRNA vaccines including components against BA.1 or BA4/5 in addition to the parental mRNA-1273 showed an equivalent protective effect against BA.5 in the lungs of mice (109) (3). We did not account for Fc-effector functions, which are important in controlling SARS-CoV-2 infection in the respiratory tract (110). Correlative studies on the therapeutic activity of purified IgG and their corresponding Fab fragments might provide some insights (4). Mouse antisera were used for the passive immunization study, and further studies on nonhuman primates and ultimately humans are needed to evaluate the translatability of our COVID-19 vaccine.

Despite these limitations, our study demonstrates the potential of live-attenuated measles vaccines and nanoparticle platforms for the development of next-generation vaccines against coronaviruses and other pathogens. By combining the robustness of the measles vaccine with the versatility of nanoparticle platforms, we can enhance the antigenicity and durability of the immune response, leading to more efficient and effective vaccines.

## MATERIALS AND METHODS

Before initiating this work, the studies described in this manuscript were granted approval by the Mayo Clinic Institutional Biosafety Committee under the research protocol Bios00000773.

### Cells and viruses

BHK cells [catalog number (Cat#) CCL-10, ATCC, Manassas, VA, USA] were maintained in Dulbecco’s modified Eagle’s medium (DMEM; Cat# SH30022.01, GE Healthcare Life, Pittsburgh, PA, USA) supplemented with 10% fetal bovine serum (FBS; Cat# 10437–028; Thermo Fisher Scientific, Waltham, MA, USA), 100 units/mL penicillin, and 100 µg/mL streptomycin (Cat# 15140122, Thermo Fisher). Vero African green monkey kidney cells expressing a membrane-anchored single-chain variable fragment specific to a hexahistidine peptide (6× HIS tag) (111) were cultured in DMEM supplemented with 5% FBS. Cells were incubated at 37°C in 5% CO_2_ with saturating humidity. The Indiana strain-based VSV expressing SARS-CoV-2 spike in place of VSV-G and trans-complemented with VSV-G has been described elsewhere (55). The recombinant measles virus based on the Moraten vaccine strain expressing firefly luciferase has been described previously (42). SARS-CoV-2 virus stocks were grown in TMPRSS2-overexpressing Vero-E6 cells maintained in DMEM supplemented with 10% FBS, 100 unit/mL penicillin, 100  µg/mL streptomycin, 1% nonessential amino acids, 3 µg/mL puromycin, and 100 µg/mL normocin. USA-WA1/2020 virus was obtained from BEI Resources (NR-52281), and Omicron BA1 virus (hCoV-19/USA/NY-MSHSPSP-PV44476/2021, GISAID: EPI_ISL_7908052) was obtained from the Mount Sinai Pathogen Surveillance Program at the Icahn School of Medicine at Mount Sinai.

### Constructs and virus rescue

The codon-optimized gene encoding Wuhan-Hu-1 (GenBank MN908947.3) was used as the basis for all SARS-CoV-2 spike constructs. The Beta variant of the SARS-CoV-2 spike protein (L18F, D80A, D215G, del242/243, R246I, K417N, E484K, N501Y, A701V) was synthesized in two fragments (Genewiz, South Plainfield, NJ, USA) and cloned into the pcDNA3.1+ expression vector (Cat# V79020, ThermoFisher Scientific, Waltham, MA, USA) using an InFusion HD Kit (Takara, Shinagawa, Tokyo, Japan). The Omicron variant of the SARS-CoV-2 spike containing the BA.1 lineage-defining amino acid changes A67V, deletion of (Δ)H69-V70, T95I, G142D, ΔV143-Y145, ΔN211, L212I, ins214EPE, T547K, D614G, H655Y, N679K, P681H, G339D, S371L, S373P, S375F, K417N, N440K, G446S, S477N, T478K, E484A, Q493R, Q498R, N501Y, Y505H, N764K, D796Y, N856K, Q954H, N969K, and L981F was synthesized and cloned into pCDNA3.1 by GenScript (Nanjing, China). All the other variants were obtained from InvivoGen (Toulouse, France). Amino acid substitutions and deletions in the SARS-CoV-2 spike protein were introduced using standard molecular biology techniques (112) and confirmed by Sanger sequencing (Genewiz). When indicated, a C-terminal thrombin cleavage site (LEVLFQGP), a “foldON” sequence (GYIPEAPRDGQAYVRKDGEWVLLSTFL) (50), and *H. pylori* NAP (GenBank accession no. WP_000846461) were also incorporated at the extreme C-terminus of the construct.

All SARS-CoV-2 spike constructs were inserted directly by InFusion cloning into the Mlu/AatII sites of the parental pSMART LC MeVvac2 (eGFP)P vector or the same plasmid comprising MeV-HΔ8/CDV-F (42). Both plasmids contain the antigenomic RNA for the measles Moraten vaccine strain. However, the latter encodes for the large-plaque-forming variant of the Canine Distemper Virus vaccine strain Onderstepoort fusion protein (CDVF_OL,_ aa 136–662) along with a full-length measles virus hemagglutinin protein (genotype H1), wherein eight antigenic sites have been modified (US Patent App. 17/518,268). The Chikungunya structural polyproteins C, E1, E2, E6, and 6K (GenBank: EU224270) from strain 37997 were amplified from the plasmid CMV/R (113) and similarly cloned into the MeV-encoding plasmid. The inserts were modified at the stop codon to comply with the paramyxovirus rule of six (114). Rescue of rMeV was carried out in cotransfected BHK cells as described previously (115).

### Viral infections and multistep growth curves

The rescued viruses were purified through plaque assay, and the single fast-growing plaques were amplified through 7 to 10 passages until sufficient titers were reached for animal experimentation. Viruses were propagated by infecting Vero cells at a multiplicity of infection (MOI) of 0.03 in viral vaccine production serum-free medium (VP-SFM, Cat#11681020, Thermo Fisher Scientific) supplemented with 2 mM L-glutamine (Cat# 25030081, Thermo Fisher). Viral titers were determined by seeding Vero cells in a 96-well plate at 10,000 cells/well and infecting them with serial 10-fold dilutions in Opti-MEM I reduced-serum medium (Cat# 31985070, Thermo Fisher). After a 90-min adsorption period, the cells were replenished with viral growth medium (DMEM + 5% FBS). The titer was visually determined 2–3 days post-infection using a microscope and calculated in terms of plaque-forming units. For virus growth analysis, Vero cells were seeded in a 6-well plate at 400,000 cells/well and infected at an MOI of 0.03. After a 1.5-h adsorption period, the inoculum was removed, the cells were washed three times with Dulbecco’s phosphate-buffered saline (Cat# MT-21–031-CVRF, Mediatech, Inc., Manassas, VA, USA), and the medium was replaced with 1 mL of VP-SFM. At various time points after infection, the cell culture fluid and cell lysates were harvested, and the virus titers were determined as described above.

### Next-generation sequencing

RNA from virus stocks was extracted with the QIAamp Viral RNA Mini Kit (Cat# 52904, QIAGEN, Hilden, Germany), and one-step cDNA synthesis was then performed with SuperScript IV RT Viral cDNA (Cat# 12594025, Thermo Fisher Scientific, Waltham, MA, USA) using the following pair of primers: F1/R3583 (5′-ACC AAA CAA AGT TGG GTA AGG ATA G-3′/5′-CAT TCA TCC TTC CTG TCG CCT AG-3′), F3409/R5488 (5′-AGC AAA GTG ATT GCC TCC CAA G-3′/5′-ATA TGG CAG AGA CGT TCA CCT TG-3′), F5380/R9560 (5′-ACA CCC GAC GAC ACT CAA C-3′/5′-GAG TTC ACG GAT CTT CCT CGT TG-3′), and F9473/R15894 (5′-GGC CCA CTC TCA TAT TCC ATA TCC-3′/5′-ATA TGG CAG AGA CGT TCA CCT TG-3′). DNA fragments were gel purified using a QIAquick Gel Extraction Kit (Cat# 28704, QIAGEN), and amplicon sequencing was performed by the Center for Computational and Integrative Biology DNA Core Facility at Massachusetts General Hospital (Cambridge, MA). Illumina-compatible adapters with unique barcodes were ligated onto each sample during library construction. Libraries were pooled in equimolar concentrations for multiplexed sequencing on the Illumina MiSeq platform with 2 × 150 run parameters. Upon completion of the NGS run, the data were analyzed, demultiplexed, and subsequently entered into an automated *de novo* assembly pipeline, UltraCycler v1.0 (Brian Seed and Huajun Wang, unpublished).

### BN-PAGE

Purified proteins were mixed with NativePAGE Sample Buffer (Thermo Fisher) and loaded into a NativePAGE 4%–12% Bis-Tris Gel (Thermo Fisher) according to the manufacturer’s instructions. The BN-PAGE gels were run for 2 h at 150 V and stained with Coomassie blue.

### Negative-stain TEM

The complex sample was diluted, and an aliquot (3 µL) was placed on a thin, carbon-coated 200-mesh copper grid that had been glow discharged. After 1 ± 0.1 min, excess solution was blotted with filter paper. The grid was washed by briefly touching the surface of the grid with a drop (30 µL) of distilled water on parafilm and blotting dry with filter paper. This touching and blotting step was performed three times, each time with a clean drop of distilled water. Three drops of 0.7% (wt/vol) uranyl formate negative stain on parafilm were then applied successively, and excessive stain was removed by blotting in the same fashion. The grid was allowed to remain in contact with the last drop of stain with the sample side down for 1–3 min in the dark before removal of excessive stain and air dried at 22°C ± 1.5°C. Images were collected with Talos L120C with an electron dose of ~40 e-/Å^2^ and magnifications of 57k× and 92k×, which resulted in pixel sizes of 0.246 and 0.152 nm at the specimen plane, respectively. Images were collected with a 4k× 4K Ceta CMOS.

### Western blot analysis

Cells grown on a 6-well plate were infected with various rMeVs at an MOI of 0.03. At 36–48 h post-infection, the supernatant was collected and filtered through a 0.45-μm pore membrane. Additionally, the cells were lysed in mammalian protein extraction reagent (Cat# 78503, Thermo Fisher Scientific) supplemented with Halt protease inhibitor cocktail (Cat# 877886, Thermo Fisher Scientific). The protein concentration was determined using a Pierce Coomassie Plus Assay Kit (Cat# 23236, Thermo Fisher), and 3 µg of cell lysate or ~20 µL of supernatant was separated on a precast 12% or 4%–12% Bis-Tris polyacrylamide gel before being transferred to a polyvinylidene fluoride membrane using an iBlot2 dry blotting system (Thermo Fisher Scientific). The blot was then probed with anti-SARS-CoV-2 spike RBD (GTX135385, GeneTex, Irvine, CA, USA), anti-SARS-CoV-2 spike (Cat# GTX632604, GeneTex), anti-MeV nucleocapsid (Cat# LS-C144599, LsBio, Seattle, WA, USA), anti-MeV hemagglutinin (116), anti-canine distemper virus fusion (Genscript), and anti-high affinity (HA) peroxidase (Cat#12013819001, Millipore Sigma, St. Louis, MO, USA) and developed with a KwikQuant Western Blot Detection Kit using a KwikQuant Imager (Kindle Bioscience LLC, Greenwich CT, USA).

### Generation of pseudovirus particles displaying SARS-CoV-2 spike and pseudovirus neutralization assay

Single-round-pseudotyped lentivirus particles were produced by the cotransfection of HEK293T cells with pHAGE-CMV-Luc2-IRES-ZsGreen-W (Cat# NR-52516, BEI), HDM-Hgpm2 (Cat# NR-52517, BEI), HDM-tat1b (Cat# NR-52518, BEI), pRC-CMV-Rev1b (Cat# NR-52519, BEI), and a SARS-CoV-2 spike plasmid as previously described (43). Virus-containing supernatants were harvested 72 h post-transfection, filtered using 0.45-µm syringe filters, divided into aliquots, and stored at −80°C until further use. For neutralization assays, the virus was diluted to yield ~50,000 relative light units (RLU)/well and incubated for 1 h at 37°C with twofold dilutions of heat-inactivated serum. HEK293T-hACE2 (Cat# NR-53821, BEI) cells were then infected in quadruplicate and lysed 72 h later using the Bio-Glo luciferase assay system (Cat# GT7940, Promega, Madison, WI, USA) to measure luciferase activity. The percentage of neutralization was calculated based on the RLU measured using the virus-only control. The half-maximal effective concentration (EC_50_) titers were calculated using a log (agonist) versus normalized response (variable slope) nonlinear function in Prism 9 for macOS (GraphPad). The first WHO International Reference panel of anti-SARS-CoV-2 immunoglobulin (NIBSC code: 20/268) was used for validation.

Alternatively, we utilized IMMUNO-CRON and IMUNO-COV v2.0 (Imanis Life Sciences) (117), which use a luciferase-encoding VSV displaying SARS-CoV-2 spike glycoproteins, to assess pseudovirus-neutralizing antibodies using Vero-hACE2 cells.

### Measles virus neutralization assay

A luciferase-based neutralization assay was used as previously reported (42). In brief, twofold serial solutions of serum samples were mixed with an equal volume of rMeV-Fluc and incubated for 1 h at 37°C. The virus-serum mix was subsequently added to Vero cells for 48 h before 50 nmol of D-luciferin (GoldBio, St. Louis, MO, USA) was added to measure luminescence. The percentage of neutralization was calculated based on the RLU measured using the virus-only control and subsequently analyzed in Prism 9 to calculate the EC_50_ using a nonsigmoidal dose-response curve. EC_50_ values were converted into milli-international units per milliliter by using the third international standard for anti-measles serum (Cat# 97/648, National Institute for Biological Standards and Control).

### Mouse immunizations

All experimental procedures were carried out in accordance with US regulations and approved by the Mayo Clinic Institutional Animal Care and Biosafety Committee (IACUC). Male and female 8- to 19-week-old mice exhibiting deficient expression of type I IFN receptor and transgenically expressing human CD46 (IFNAR^−/−^-CD46Ge) (118) were bred in-house under specific pathogen-free conditions and regularly controlled by animal care takers and institutional veterinarians for general signs of well-being. Animals were maintained at a constant temperature of 22°C–25°C and relative humidity of 40%–70% with a 12-h light/dark cycle and were provided food and water *ad libitum*. For the experiments, animals were randomized for age- and sex-matched groups, and no statistical consideration was performed. Given the limited supply of animals stemming from shelter-in-place regulations, the number of animals per experiment was subjected to fluctuation. These animals were vaccinated intraperitoneally with the indicated plaque-forming units of recombinant viruses or purified SARS-CoV-2 spike protein adjuvanted with aluminum hydroxide (alum, 2% alhydrogel adjuvant, Cat# vac-alu-250, InvivoGen, San Diego, CA, USA). A prime-boost vaccination regimen was used, and serum samples were collected before the vaccination booster was administered and at the end of the study. At this point, the mice were euthanized, and splenocytes were harvested for study of the cellular immune responses. All serum samples were heat inactivated for 30 min at 56°C before humoral immune responses were assessed.

### Passive serum transfer

Transgenic K18-hACE2 mice (strain #:034860) were purchased from Jackson Laboratories and housed in a temperature-controlled vivarium with a 12-h day/night regime with water and food provided *ad libitum*. All experimental procedures were approved by the IACUC of the Icahn School of Medicine at Mount Sinai. For passive immunization, 150 µL of pooled serum was passively introduced by intraperitoneal injection 2 h before infection. The mice were infected intranasally with 10^4^ pfu virus diluted in PBS, which was administered in 50 µL divided between both nostrils under mild ketamine/xylazine sedation (75 mg/kg ketamine; 7.5 mg/kg xylazine). Mice were monitored daily, and body weights were recorded. On day 3 or 5 post-infection, mice were euthanized via intraperitoneal injection of sodium pentobarbital (292.50 mg/kg). Lungs and nasal turbinates were isolated aseptically in 500 µL of PBS and homogenized for further use. Homogenates from lung and nasal turbinates were titrated to determine the virus load by a plaque assay using TMPRSS2-expressing Vero cells, as previously described (119).

To passively immunize Ifnar^−/−^CD46Ge mice, we administered pooled antiserum containing 400 mIU of measles nAbs from mice that had previously been vaccinated. We administered the injection through the same route precisely 2 h prior to rMeV vaccination.

### Recombinant antigens

Recombinant SARS-CoV-2 proteins produced in a baculovirus system were commercially obtained from Sino Biological as follows: S1 + S2 ectodomain (Cat# 40589-V08B1), S1 (Cat# 40591-V08B1), RBD (Cat# 40592-V08B), S2 (Cat# 40590-V08B), and nucleocapsid (Cat# 40588-V08B). Trimeric SARS-CoV-2 spike and spike-*H. pylori* NAP proteins (SARS-CoV-2S6p3 and SARS-CoV-2S6p312, respectively) were produced upon transient expression in Expi293F cells (Thermo Fisher). Clarified supernatants were purified by affinity chromatography using an anti-HA matrix (Cat# 11 815 016 001, Millipore Sigma) preequilibrated with 20 mM Tris, 0.1 M NaCl, and 0.1 mM EDTA, pH 7.5 (equilibration buffer). The column was washed with equilibration buffer containing 0.05% Tween 20, and then elution was performed with 1 mg/mL HA synthetic peptide (Cat# 26184, Thermo Fisher) per the manufacturer’s instructions. The fractions containing the eluted proteins were combined, concentrated, and dialyzed against Dulbecco’s PBS (Cat# 25–508, Genesee Scientific) using a Pierce protein concentrator with a 10-kDa molecular weight cutoff (Cat# 88516, Thermo Fisher). The HA matrix was regenerated with 20 volumes of 0.1 M glycine, pH 2.0 (Cat# SC295018, Santa Cruz Biotechnology) and re-equilibrated before the next purification round. The protein concentration was determined using a Pierce 660 Protein Assay Kit (Cat# 22662, Thermo Fisher). SARS-CoV-2S6p312 was purified after SARS-CoV-2S6p3 and stored at −80°C until use. Recombinant Chikungunya virus E2 protein produced in a baculovirus system was commercially obtained from Sino Biological (Cat#40440-V08B).

### Antigen-binding enzyme-linked immunosorbent assay

IgG binding to SARS-CoV-2, MeV antigens, or CHKV E2 was measured by ELISA using clear flat-bottom Immuno nonsterile 96-well plates (Cat# 442404, Thermo Fisher Scientific) coated overnight at 4°C with 100 ng of recombinant SARS-CoV-2 proteins or CHKV E2 protein or 1 µg of MeV bulk antigen (Cat# BA102VS, Institut Virion\Serion GmbH, Würzburg, Germany) in 50 mM carbonate-bicarbonate buffer, pH 9.6. The plates were washed and blocked with 2% bovine serum albumin in PBS for 2 h at room temperature (RT). The plates were washed again and incubated with serial dilutions of mouse serum for 1 h at 37°C. The plates were washed three times with PBS with 0.05% Tween 20 and then incubated for 1 h at RT with horseradish peroxidase-conjugated anti-mouse IgG (1:5,000, Cat# 62–6520, ThermoFisher Scientific), IgG1 (1:5,000, Cat# 115–035-205, Jackson ImmunoResearch), or IgG2a (1:5,000, Cat# 115–035-206, Jackson ImmunoResearch) secondary antibodies. After the final wash, the plates were developed using 50 µL of 1-Step Ultra TMB (3,3′,5,5′-tetramethylbenzidine; Thermo Fisher Scientific), and the reaction was stopped with an equal volume of 2 M sulfuric acid before the optical density (OD) was read at 405 nm using an Infinite M200Pro microplate reader (Tecan). The endpoint titers of serum IgG responses were determined as the dilution at which the OD exceeding the average of the OD values plus three standard deviations of that of pooled negative serum samples was observed. Alternatively, anti-SARS-CoV-2 binding IgG levels were reported in units of microgram per milliliter based on a standard curve that was generated using a SARS-CoV-2 spike nAb (Cat# 40595-MM57, Sino Biological).

### T-cell responses to viral antigens

IFN-γ enzyme-linked immunospot assays were carried out using mouse splenocytes to assess T-cell responses against MeV and SARS-CoV-2 peptides. Briefly, 5 × 10^5^ isolated splenocytes were cocultured with different stimuli in 200 µL of RPMI-10% FBS complete medium for 48 h on IFN-γ-coated plates (Cat# EL485, R&D systems, Minneapolis, USA). Fifteen-mer overlapping peptides from SARS-CoV-2 spike glycoprotein (Cat# PM-WCPV-S-1, JPT peptides, Berlin, Germany) and MeV-nucleoprotein (Genscript, NJ, USA) were used to stimulate splenocytes at 5 µg/mL. As a positive control, a phorbol myristate acetate/ionomycin cell stimulation cocktail (Biolegend, San Diego, CA, USA) was used at 2.5 µL/mL, and as a negative control, splenocytes were stimulated with an equivalent DMSO concentration (0.8%). At 48 h post-incubation, the plates were developed in accordance with the manufacturer’s instructions. The developed IFN-γ spots were counted with an automated ELISpot reader (CTL Analyzers LLC, USA). Each spot represented a single reactive IFN-γ-secreting T cell.

### Measurement of Th1/Th2 cytokines using *ex vivo* stimulation of splenocytes with antigen peptides

Frozen splenocytes were thawed and incubated with 50 µg/mL DNase1 (Cat# 10104159001, Roche) for 5 min at 37°C. The cells were then washed twice and resuspended in RPMI-1640 medium with 10% (vol/vol) heat-inactivated FBS. Splenocytes (1 × 10^6^/well in 96-well plates) were stimulated for 24 h with 15-mer overlapping peptides from SARS-CoV-2 spike glycoprotein (Cat no# PM-WCPV-S-1, JPT Peptide Technologies GmbH) or VSV-N (Genscript) at a concentration of 2.5 µg/mL. Supernatants were collected, centrifuged at 1,800 RPM for 5 min, and stored at −80°C until analysis. Supernatants were then analyzed for the expression of IFN-γ, IL-6, IL-18, GM-CSF, IL-1β, IL12p70, IL-13, IL-2, IL-4, TNF-α, and IL-5 cytokines using a Mouse Cytokine 11-Plex Antibody Bead Kit (Th1/Th2 Cytokine 11-Plex Mouse ProcartaPlex Panel, Cat No# EPX110-20820-901, Thermo Fisher). Sample preparation, along with kit standards, detection antibody, and streptavidin-phycoerythrin, was carried out according to the manufacturer’s instructions. Cytokine bead fluorescence intensity was measured using the Luminex 200 system (Luminex Corp., Austin, TX, USA), and data were quantitated with xPONENT software.

### Statistical analysis

Statistical analyses were performed with GraphPad Prism version 9.1.0 for Mac OS 10.15.7. Significant differences among groups were determined as described in the figure legends.

## Data Availability

All data needed to evaluate the conclusions in the paper are present in the paper and/or the Supplementary Materials. All other data are available from the lead contact. Materials are available under a material transfer agreement.

## SUPPLEMENTAL MATERIAL

Figure S1 mbio.02928-23-s0001.tif Consistency of neutralization titers for SARS-CoV-2 spike-pseudotyped lentivirus.

Figure S2 mbio.02928-23-s0002.tif Biochemical characterization of SARS-CoV-2 proteins used in the immunization studies.

Figure S3 mbio.02928-23-s0003.tif Anti-MeV nAbs impair the heterologous immune response to CHIKV E2 proteins.

Figure S4 mbio.02928-23-s0004.tif Expression of SARS-CoV-2 spike-based constructs from the rMeV-MR vector.

Figure S5 mbio.02928-23-s0005.tif Comparison of the immune response elicited by rMeV and VSV vectors expressing SARS-CoV-2 spike.

Figure S6 mbio.02928-23-s0006.tif Preexisting measles immunity impacts neutralizing and T-cell responses from a measles Moraten-CoV-S6p312 vaccine candidate.

Supplemental legends mbio.02928-23-s0007.docx Legends for Fig. S1 to S6.

ASM does not own the copyrights to Supplemental Material that may be linked to, or accessed through, an article. The authors have granted ASM a non-exclusive, world-wide license to publish the Supplemental Material files. Please contact the corresponding author directly for reuse.
