# Supplementary figures and images for: Surface-modified measles vaccines encoding oligomeric, prefusion-stabilized SARS-CoV-2 spike glycoproteins boost neutralizing antibody responses to Omicron and historical variants, independent of measles seropositivity
Source: mBio. 2024 Jan 9;15(2):e02928-23. doi: 10.1128/mbio.02928-23 (PMC10865805; doi:10.1128/mbio.02928-23)

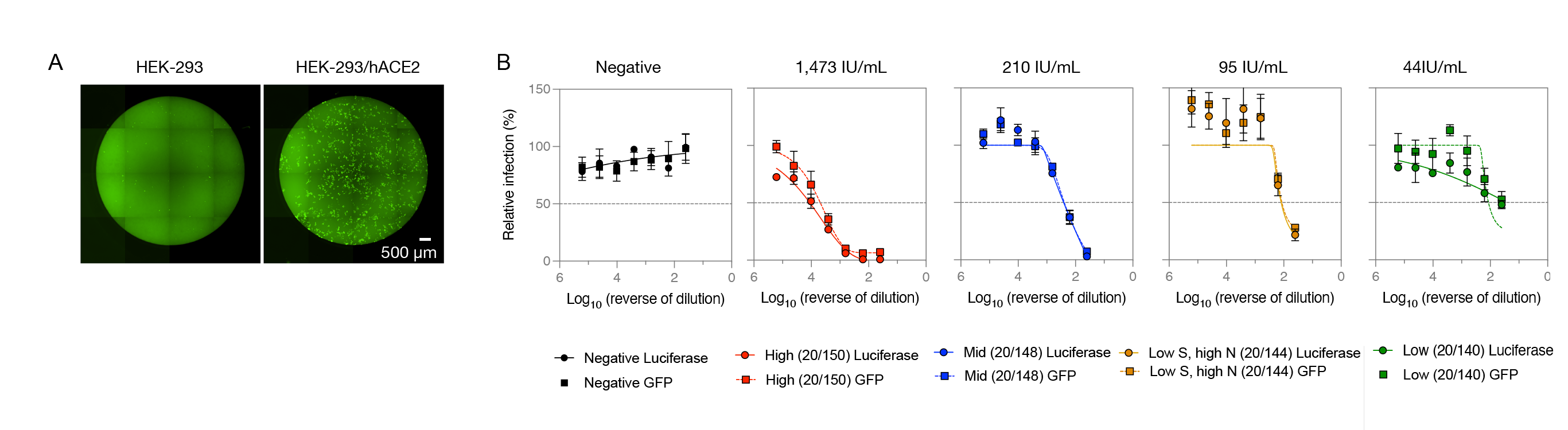

Supplement: Figure S1 — Consistency of neutralization titers for SARS-CoV-2 spike-pseudotyped lentivirus. [file mbio.02928-23-s0001.tif]

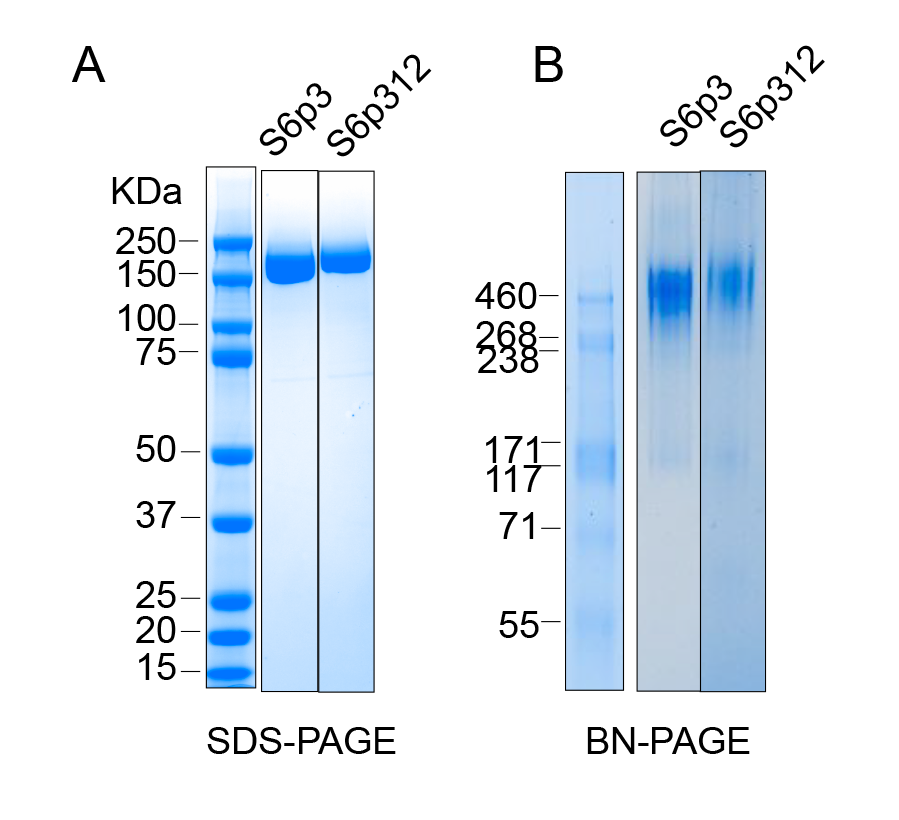

Supplement: Figure S2 — Biochemical characterization of SARS-CoV-2 proteins used in the immunization studies. [file mbio.02928-23-s0002.tif]

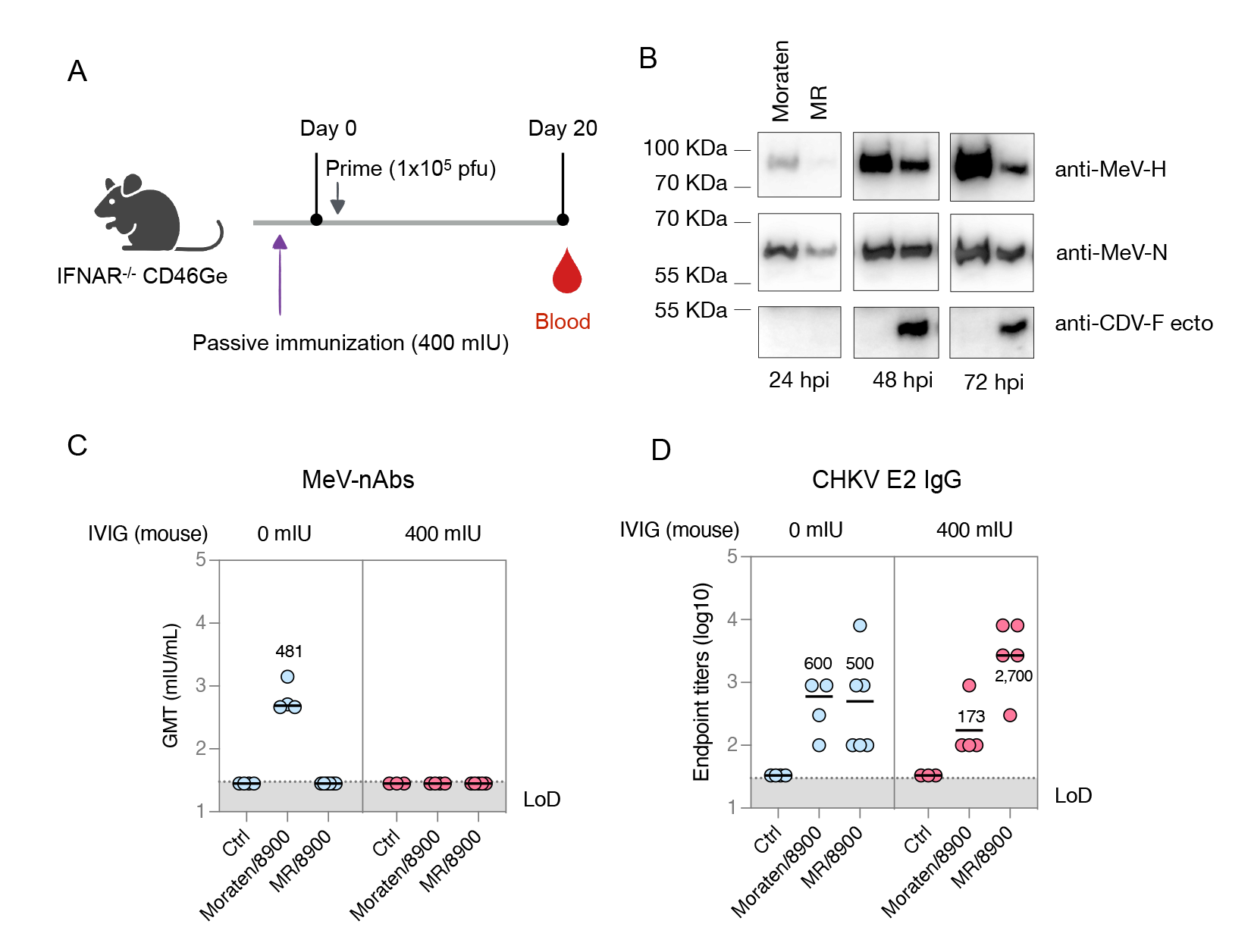

Supplement: Figure S3 — Anti-MeV nAbs impair the heterologous immune response to CHIKV E2 proteins. [file mbio.02928-23-s0003.tif]

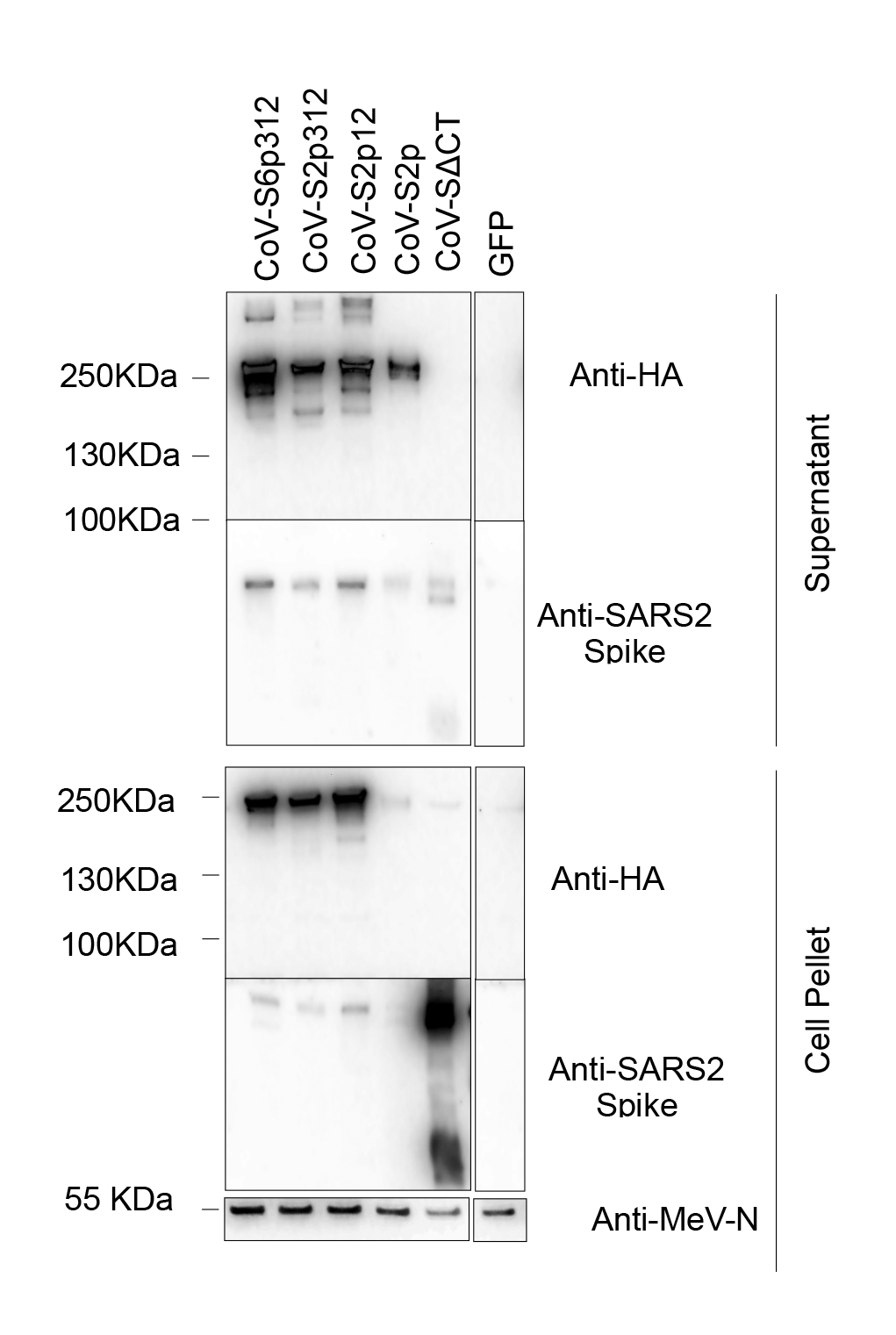

Supplement: Figure S4 — Expression of SARS-CoV-2 spike-based constructs from the rMeV-MR vector. [file mbio.02928-23-s0004.tif]

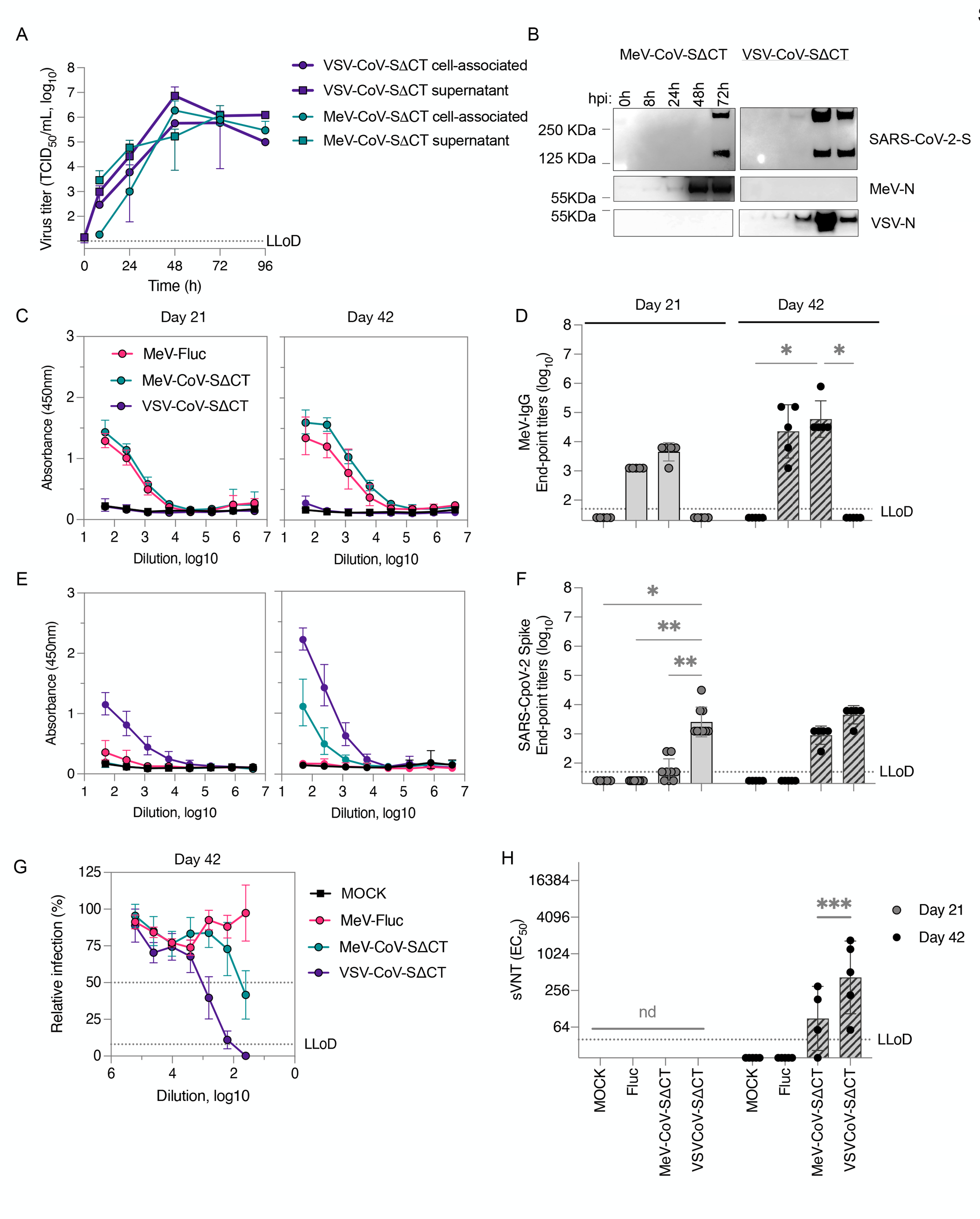

Supplement: Figure S5 — Comparison of the immune response elicited by rMeV and VSV vectors expressing SARS-CoV-2 spike. [file mbio.02928-23-s0005.tif]

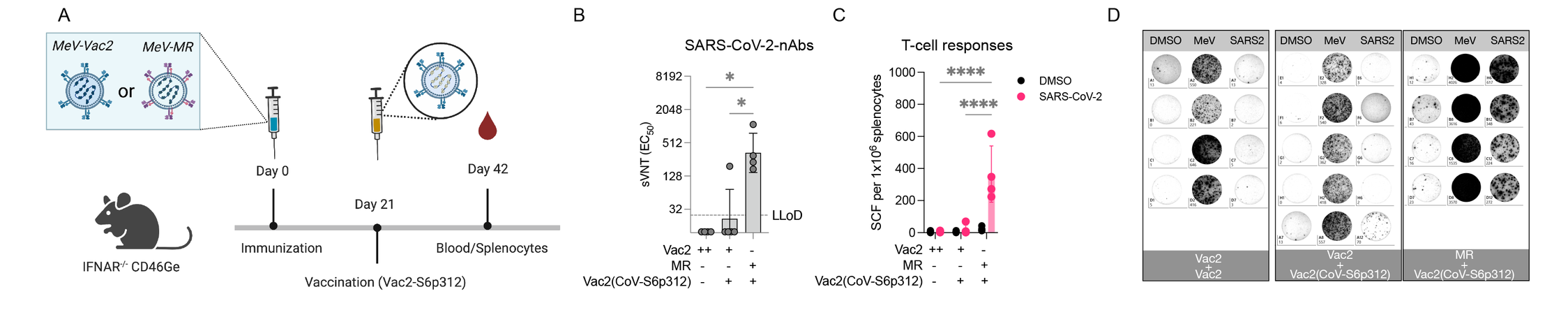

Supplement: Figure S6 — Preexisting measles immunity impacts neutralizing and T-cell responses from a measles Moraten-CoV-S6p312 vaccine candidate. [file mbio.02928-23-s0006.tif]
