# Supplementary material for: Surface-modified measles vaccines encoding oligomeric, prefusion-stabilized SARS-CoV-2 spike glycoproteins boost neutralizing antibody responses to Omicron and historical variants, independent of measles seropositivity
Source: mBio. 2024 Jan 9;15(2):e02928-23. doi: 10.1128/mbio.02928-23 (PMC10865805; doi:10.1128/mbio.02928-23)
Supplement: Supplemental legends — Legends for Fig. S1 to S6. [file mbio.02928-23-s0007.docx]

Supplementary Materials for

Surface-modified measles vaccines encoding oligomeric, prefusion-stabilized SARS-CoV-2 spike glycoproteins boost neutralizing antibody responses to Omicron and historical variants, independent of measles seropositivity

Miguel Á. Muñoz-Alía et al.

*Corresponding author. Email: [mamunoz.alia@vyriad.com](mailto:mamunoz.alia@vyriad.com)

**This PDF file includes:**

Figs. S1 to S6

Fig. S1.Consistency of neutralization titers for SARS-CoV-2 spike-pseudotyped lentivirus. (A) The transduction efficiency of SARS-CoV-2 spike-pseudotyped lentiviral particles (LVs) was assessed using both HEK293 and hACE2-HEK293 cells, and transduction was visualized three days later using a Celigo Imaging Cytometer. (B) Pseudovirus neutralization assays were performed using luminescence and fluorescence-based methods with SARS-CoV-2/LVs and the first World Health Organization International reference panel for anti-SARS-CoV-2 immunoglobulins. The transduction units (TU) were determined directly by the Celigo instrument or indirectly by measuring luciferase activity. The relative infection was calculated by dividing either TU or luminosity in the presence of human serum by the values obtained in its absence and then converting to a percentage for each serum dilution. Data points denote the mean ± standard deviation of an experiment performed in quadruplicate, and data were fitted by nonlinear regression analysis via Graph Pad Prism.

Fig. S2. Biochemical characterization of SARS-CoV-2 proteins used in the immunization studies. (A) SDS‒PAGE analysis of purified SARS-CoV-2 proteins using a 4-12% Bis-Tris gel and Coomassie staining. (B) BN-Native electrophoresis of purified SARS-CoV-2 proteins using a 4-16% Bis-Tris gel followed by Coomassie staining.

**Fig. S3. Anti-MeV nAbs impair the heterologous immune response to CHIKV E2 proteins**. **(A)** Schematic representation of the experimental design where IFNAR^−/−^-CD46Ge mice were pretreated with 400 mIU of mouse anti-MeV nAbs and then vaccinated with MeV Moraten or MeV-MR vectors encoding CHIKV structural proteins. Blood samples were collected 20 days post-vaccination. **(B)** Western blot analysis confirmed the expression of MeV and MeV-MR virus-specific proteins. **(C)** Generation of MeV nAbs was tested in the presence or absence of passive immunity. **(D)** IgG binding responses to CHIKV E2 proteins were measured.

Fig. S4. Expression of SARS-CoV-2 spike-based constructs from the rMeV-MR vector. Vero cells were infected with various SARS-CoV-2 Spike-based measles vector constructs at an MOI of 0.03, and cell lysates and supernatants were collected two days post-infection. Western blotting was performed using specific antibodies to detect the expressed proteins. Molecular weight markers are shown for reference.

# **FIG S5. Comparison of the immune response elicited by rMeV and VSV vectors expressing SARS-CoV-2. (A)** Multistep growth kinetics of rMeV- (Moraten backbone) and VSV-CoV-2SΔCT in Vero cells were analyzed after infection at an MOI of 0.03. **(B)** Time-course Western blot analysis of infected Vero cells. **(C to F)** ELISA was used to determine MeV- **(C and D)** and SARS-CoV-2-spike **(E and F)** binding IgG using serum from mice vaccinated once or twice (days 0 and 21) with rMeV expressing either CoV-SΔCT or firefly luciferase (Fluc) and VSV-CoV-SΔCT. The results are presented as the absorbance over the inverse of the serum dilution (left two panels) or reciprocal endpoint dilution (outer right panel) with lines and dots depicting geometric mean ± geometric standard deviation. **(G and H)** Neutralizing antibody responses against SARS-CoV-2 were determined using pseudotyped viruses expressing the D614G spike protein. "Nd" indicates "not determined". Statistical significance was calculated by two-way ANOVA (*, p < 0.05; **, p < 0.01; ***, p < 0.0005).

**FIG S6. Preexisting measles immunity impacts neutralizing and T-cell responses from a measles Moraten-CoV-S6p312 vaccine candidate.** **(A)** IFNAR^−/−^-CD46Ge mice were vaccinated with MeV Moraten (vac2) or MeV-MR vectors and subsequently with vac2-CoV-S6p312 (Moraten backbone). Three weeks later, serum and spleens were collected. **(B)** Neutralizing responses to SARS-CoV-2 pseudovirus were recorded for different treatment groups. The control group (++) comprised animals that received two doses of vac2 virus. **(C)** The T-cell responses against SARS-CoV-2 spike (S2 subdomain) were determined by ELISPOT and represented as individual dots. The bars and error bars indicate the geometric mean ± geometric standard deviation. **(D)** Images show the detection of IFN-γ-generating cells in response to overlapping peptides that span the SARS-CoV-2 spike (S2 subdomain), MeV-nucleocapsid peptides, or control medium (DMSO). Statistical analysis was performed using one-way ANOVA (Panel B; *, p<0.05) or two-way ANOVA with Bonferroni’s multiple comparison test (Panel C; ***, p<0.0001).
